# Genome-wide analysis of AAAG and ACGT *cis-*elements in *Arabidopsis thaliana* reveals their involvement with genes downregulated under jasmonic acid response in an orientation independent manner

**DOI:** 10.1093/g3journal/jkac057

**Published:** 2022-03-18

**Authors:** Zaiba H Khan, Siddhant Dang, Mounil B Memaya, Sneha L Bhadouriya, Swati Agarwal, Sandhya Mehrotra, Divya Gupta, Rajesh Mehrotra

**Affiliations:** 1 Department of Biological Sciences, Birla Institute of Technology and Science-Pilani, Zuarinagar, Goa 403726, India; 2 Department of Biological Sciences, Birla Institute of Technology and Science-Pilani, Pilani, Jhunjhunu, Rajasthan 333031, India; 3 Department of Computer Science and Information Systems, Birla Institute of Technology and Science-Pilani, Zuarinagar, Sancoale, Goa 403726, India; 4 Faculty of Bioscience, Institute of Biosciences and Technology, Shri Ramswaroop Memorial University, Barabanki, Uttar Pradesh 225003, India

**Keywords:** Dof, bZIP, *cis-*regulatory elements, transcription factor, promoter

## Abstract

*Cis-*regulatory elements are regions of noncoding DNA that regulate the transcription of neighboring genes. The study of *cis*-element architecture that functions in transcription regulation are essential. AAAG and ACGT are a class of *cis*-regulatory elements, known to interact with Dof and bZIP transcription factors respectively, and are known to regulate the expression of auxin response, gibberellin response, floral development, light response, seed storage proteins genes, biotic and abiotic stress genes in plants. Analysis of the frequency of occurrence of AAAG and ACGT motifs from varying spacer lengths (0–30 base pair) between these 2 motifs in both possible orientations—AAAG _(N)_ ACGT and ACGT _(N)_ AAAG, in the promoters and genome of *Arabidopsis thaliana* which indicated preferred orientation of AAAG _(N)_ ACGT over ACGT _(N)_ AAAG across the genome and in promoters. Further, microarray analysis revealed the involvement of these motifs in the genes downregulated under jasmonic acid response in an orientation-independent manner. These results were further confirmed by the transient expression studies with promoter-reporter cassettes carrying AAAG and ACGT motifs in both orientations. Furthermore, cluster analysis on genes with AAAG _(N)_ ACGT and ACGT _(N)_ AAAG motifs orientations revealed clusters of genes to be involved in ABA signaling, transcriptional regulation, DNA binding, and metal ion binding. These findings can be utilized in designing synthetic promoters for the development of stress-tolerant transgenic plants and also provides an insight into the roles of these motifs in transcriptional regulation.

## Teaser

*Cis*-regulatory elements are regions of non-coding DNA that regulate the transcription of neighboring genes. AAAG and ACGT are classes of *cis*-regulatory motifs that interact with Dof and bZIP transcription factors, respectively. Here, Khan *et al*. use *in silico* approaches to perform a genome-wide promoter analysis, determining the frequency and patterns of occurrence of these motifs in *Arabidopsis thaliana*. Their findings will aid the design of synthetic promoters for the development of stress-tolerant transgenic plants.

## Introduction

*Cis*-regulatory elements are short, functional noncoding DNA sequences, clustered within the promoter region of genes (Wray *et al.* 2003). These motifs play a key role in regulating the spatio-temporal expression of downstream genes at the transcriptional level. By providing binding sites to the sequence-specific cognate transcription factors (TFs), they lead to the activation or repression of the gene (Stormo 2000). The overall binding specificity of TFs to these short sequences depends upon a variety of factors including distance from the transcription start site (TSS), inter-motif distance, spacer sequence, copy number, and orientation related to the gene. Two motifs that we studied in this present study are AAAG and ACGT. It has been shown that the Dof binding proteins bind to AAAG sequences and the flanking sequences have limited effects (Yanagisawa 2002). However, this is not the case with ACGT core sequences, wherein flanking sequences influence the gene expression (Izawa *et al.* 1993). Dof proteins are shown to be involved in a multitude of functions. The role includes stress responses (Kang and Singh 2000; Ma *et al.* 2015a; Wang *et al.* 2017a), light responses (Yanagisawa and Sheen 1998), response to auxin (Baumann *et al.* 1999), gibberellins (Washio 2001), tissue-specific expression in endosperms (Vicente-Carbajosa *et al.* 1997; Mena *et al.* 1998), floral development (Rojas-Gracia *et al.* 2019). bZIP family of TFs is involved in abscisic acid (ABA) response (Zong *et al.* 2016), salicylic acid (SA) response (Li *et al.* 2019), auxin (Weiste *et al.* 2017), anaerobiosis (Sell and Hehl 2004), jasmonic acid (JA) (Liu *et al.* 2019), and UV responsiveness (Binkert *et al.* 2014).

bZIP and Dof interaction has been shown in the promoter of a seed storage protein gene (Zein) from maize. This promoter contains P-box (Yanagisawa 2000; Mehrotra *et al.* 2014) a highly conserved 7-bp sequence *cis*-element with TGTAAAG conserved sequence and OCS element (ACGT) which are binding sites for Dof (P-box binding factors) and bZIP (O2) TFs, respectively (Chen *et al.* 1996). Members of the bHLH family of TFs such as MYC2, MYC3, and MYC 4 are also known to interact with Dof, These interactions are crucial for mediating JA transcriptional responses in *Arabidopsis thaliana* (Fernández-Calvo *et al.* 2011; Zhuo *et al.* 2020). The motivation for this study is to decipher the interaction between Dof and bZIP TFs if any in stress gene regulation through *cis*-element binding. In this study, we performed a genome-wide and a promoter-wide analysis by employing various in silico approaches to determine the frequency of occurrence and to identify patterns of occurrence of these motifs in tandem gradually increasing spacers from 0 to 30 base pair (bp), in both possible orientations-AAAG _(N)_ ACGT and ACGT _(N)_ AAAG, in the genome of model plant *A.* *thaliana.* Optimum inter-motif distance has great importance as promoter activation by ACGT is differentially regulated by the spacing between the 2 motifs (Mehrotra and Mehrotra 2010). Furthermore, a TF binding site (TFBS) analysis was performed to determine the binding sites for several TFs using ConSite: The functional software (Sandelin *et al.* 2004). Following this, microarray data analysis was performed using the genes upregulated/downregulated by hormones (ABA, auxin, ethylene, gibberellin, JA, and SA), environmental conditions (at baseline growth temperature, disease, drought, low water potential, at optimum photosynthetic temperature, salt, 20% inhibition from optimum photosynthetic temperature, 30% inhibition from optimum photosynthetic temperature) with the view to uncover the pattern of AAAG and ACGT distribution in the promoter of stress-induced genes (Mehrotra *et al.* 2013). The data suggested the involvement of AAAG and ACGT motifs in genes downregulated during JA responses in either orientation. Further, transient expression studies have been carried out to confirm the in silico findings. The gene ontology (GO) and cluster analysis revealed clusters of genes to be involved in ABA signaling, transcriptional regulation, DNA binding, and metal ion binding. The data obtained in this study highlights the importance of spacer lengths and orientation which can be used for designing inducible promoters. It also provides an understanding of the involvement of ACGT and AAAG sequences in the transcriptional regulation of stress-induced genes.

## Materials and methods

### DNA sequence retrieval

Genome-wide and promoter analysis was performed in *A.* *thaliana* genes (27,416) and promoters. For genome-wide analysis, the DNA sequence for all chromosomes was retrieved from TAIR (The Arabidopsis Information Resource, version 10; Huala *et al.* 2001; Lamesch *et al.* 2012). Further, Python code was used to extract 1 kb upstream promoter region of all genes across 5 chromosomes of *A.* *thaliana*.

The spacer frequency analysis was aimed at searching for co-occurring AAAG and ACGT elements of varying length increasing from 0 to 30 bp in the entire genome and 1 kb regions of promoters. We calculated the enrichment of these motifs by dividing frequency with the genomic coverage in genome and promoter regions respectively from 0 to 30 bp spacer in both orientations. As it has been hitherto reported that binding of corresponding TFs to the DNA sequence are usually spaced within 25–30 bp, we restricted our analysis to 30 bp spacer distance between AAAG and ACGT motifs (Mehrotra *et al.* 2013). The nucleotide sequence of each spacer between AAAG and ACGT motifs was extracted and the total number of occurrences for each spacer length was determined.

### Spacer sequence analysis

Further, another Python code was executed on the extracted sequences to determine the frequency of occurrence of AAAG and ACGT motifs in tandem, for varying spacer lengths from 0 to 30 bp, in both possible orientations, i.e. AAAG _(N)_ ACGT and ACGT _(N)_ AAAG. Further, exact nucleotide sequences, for all spacer lengths from 0 to 30 bp of AAAG _(N)_ ACGT and ACGT _(N)_ AAAG, were also determined. Since flanking sites of the core sequence is essential for binding of TFs so we performed an analysis on flanking sites of the motifs: TAAAG and GACGTC, TGTAAAG and GACGTC in both the possible orientation. A comparison of core motifs to a longer motif was done in order to identify whether flanking sequences are predominating or not. There are many reports which show that any flanking sites can alter the binding site of TFs (Guiltinan *et al.* 1990; Morin *et al.* 2006). Further, to test the significance of AAAG _(N)_ ACGT and ACGT _(N)_ AAAG sequences, we used 12 random combinations of the 2 sequence sets: AAGA, AGAA, GAAA and CATG, GCAT, GTAC, TCAG as controls generated by shuffling AAAG and ACGT in all possible ways. Using the PLACE database, we ensured that each of these tetramer sequences is not conserved *cis*-element themselves (Higo *et al.* 1999). By performing a similar analysis on each control sequence, we compared the frequency of the AAAG _(N)_ ACGT motif with the corresponding frequency of control sequences for the same *N* = 0–30 bp.

Frequency of occurrence of exact sequences for all spacers 0–30 bp were extracted, and consensus sequences for all spacer lengths were generated by calculating percentage A, C, G, T content at each position in all spacers. After calculating the percentage content of the 4 nucleotides, thresholds on percentage content are set for considering a nucleotide at a particular position to be conserved. Since the % G/C content of *A.* *thaliana* is about 36% (Mishra *et al.* 2009), we have taken the thresholds as 25% for G/C and 40% for A/T. If the frequency of G or C was more than 25% of the GC-content, we assumed that G or C is the preferred base. Similarly, if A or T had a frequency of occurrence of more than 40% at that particular position, then A or T is the preferred base.

### Statistical analysis

A paired Student’s *t*-test was conducted using the standard protocol to test the statistical significance of the results (McDonald 2014). For this, the frequency of occurrence from 0 to 30 bp between AAAG_(N)_ACGT and ACGT_(N)_AAAG were compared with various combinations of sequences AAGA, AGAA, GAAA, CATG, GCAT, GTAC, TCAG, and paired student t-test was applied to test the statistical significance of the sequences.

### TFBS analysis

We wanted to decipher the biological significance of the *cis*-elements along with its spacer contributing to the high level of expression at the transcriptional level. For this, TF binding site analysis was performed using ConSite, a tool used to predict the TF binding site (TFBS) on regulatory elements (Sandelin *et al.* 2004). Prediction of TF binding sites on highly occurring spacer sequences between AAAG and ACGT motifs obtained through our spacer sequence analysis was carried out by prefixing a 139 nucleotide-long minimal promoter sequence (MPS) before those sequences (Kiran *et al.* 2006). Generally, MPS is a minimal promoter sequence that is merely required to achieve a basal level of gene expression in vivo. Other sequences along with the MPS are required to achieve a high level of gene expression. The sequence of MPS is as follows:

TCACTATATATAGGAAGTTCATTTCATTTGGAATGGACACGTGTTGTCATTTCTCAACAATTACCAACAACAACAAACAACAAACAACATTATACAATTACTATTTACAATTACATCTAGATAAACAATGGCTTCCTCC. TFBSs present on the highly occurring spacer sequences between AAAG**_(0_**_**–**__**30 bp)**_ ACGT and ACGT_**(0**__**–**__**30 bp)**_AAAG motifs and MPS were compared and contrasted to check for binding suitability for TFs. The cut-off score for binding specificity was set to 80%.

### Functional analysis

Microarray data of genes upregulated and downregulated under various environmental conditions (at baseline growth temperature, disease, drought, low water potential, at optimum photosynthetic temperature, salt, 20% inhibition from optimum photosynthetic temperature, 30% inhibition from optimum photosynthetic temperature) or by hormones (ABA, auxin, ethylene, gibberellin, JA, and SA) were retrieved from EBI expression atlas (Kapushesky *et al.* 2010) to analyze whether genes containing multiple core AAAG and ACGT *cis*-elements were upregulated or downregulated during these conditions. By comparing genes regulated by a condition with genes containing multiple AAAG _(N)_ ACGT and ACGT _(N)_ AAAG elements, we calculated the likelihood of occurrence by the following formula:

Likelihood   of   occurrence=X/Y  for  a  particular  spacer  length(N = 0–30  bp)

where X = (A ∩ B)/B and Y = *P*(A);

A = event that a given gene is regulated (up/down) by a particular condition B = event that a given gene contains multiple AAAG-ACGT elements separated by *N* bps. Further, we calculated the overall likelihood of occurrence for each condition (for *N* = 0–30 bp). We set the threshold value as > 1.25 for the likelihood of occurrence for all conditions. This was followed by our previous work (Mehrotra *et al.* 2013).

### GO and cluster analysis on the basis of functional characteristics of genes

We intended to perform GO analysis on the genes downregulated under the JA condition with ACGT_(N)_AAAG and AAAG_(N)_ACGT motifs within their promoters using DAVID (The **D**atabase for **A**nnotation, **V**isualization and **I**ntegrated **D**iscovery, v6.8) tool (Huang *et al.* 2009b). We further refined the data by doing functional clustering on the data. The functional annotation clustering on GO term analysis results was conducted using the DAVID (Huang *et al.* 2009a). Each gene was represented using gene set enrichment analysis (GSEA) to identify GO: biological processes, molecular functions, and cellular components. For each orientation of motifs, clusters were created based on the similarity in functional analysis. Within each cluster, the genes were further sub-clustered based on the similarity between their category and term functions. An enrichment score was fetched for each cluster. Since within sub-clusters several genes were repeated, we took only unique gene identifiers and visualized the clusters. Similarly, cluster analysis of genes based on spacer sequence analysis was also conducted. As discussed above, spacer sequence analysis was conducted on genes for varying spacer lengths from 0 to 30 bp in both AAAG _(N)_ ACGT and ACGT _(N)_ AAAG orientations. We further performed multidimensional clustering on 74 genes downregulating under JA in both orientations based on their spacer lengths. We found 34 and 33 such genes in AAAG _(N)_ ACGT and ACGT _(N)_ AAAG orientations respectively. We also discard the spacers that have 0 occurrences across all genes making them insignificant for the clustering. We found 10 and 4 such spacers in AAAG _(N)_ ACGT and ACGT _(N)_ AAAG orientations, respectively. We used the K-means clustering algorithm to fit or train our model (Kanungo *et al.* 2002). Since the number of K clusters in K-means is user-generated and sometimes can lead to poor clustering if not selected based on the data. Therefore, we use Silhouette Score (Gat-Viks *et al.* 2003) to identify the number of optimal clusters. The silhouette score is a well-known and commonly practiced metric to identify the value of K. It uses the concept of mean inter-similarity and mean intracluster similarity and ranges from −1 to 1. It iteratively computes these distances/similarities for different values of K. The aim is to increase the intracluster similarity (cohesion) and decrease the inter-similarity (representing the separation from genes in other clusters).

### Preparation of promoter-reporter cassette

A minimal promoter *Pmec* sequence containing a TATA-box, a TSS, and the reporter gene *gusA* cloned in the plasmid pUC19 was used. A random sequence of 50 nucleotides (GGATCCGGCTATGGCGGAGCAAGATTCACTCTGCGAGGCCAAAGCTTACCCCGGAAGGATCC), was cloned at the *BamH1* site of the *Pmec*. Further, this promoter-reporter cassette was cloned in the pBluescript SK (±) Phagemid (Stratagene, USA). Upstream of the 50 random nucleotide sequence, different combinations of the AAAG and ACGT motifs: AAAG_(N)_AAAG; *N* = 0, 5, 25 bp, ACGT_(N)_ACGT; *N* = 0, 5, 25 bp, AAAG_(N)_ ACGT; *N* = 0, 5, 25 bp ACGT_(N)_AAAG; *N *= 0, 5, 25 bp were inserted at the *Xba*I site (Supplementary Table 1 and Supplementary Fig. 1). The AAAG/ACGT–Pmec-*gusA* cassettes were coated on gold microparticles and bombarded onto tobacco leaves at 1100 psi, using a biolistic gun (Bio-Rad PDS-1000/He).

### Transient expression studies under JA

To study the expression of the reporter gene *gusA* using different minimal promoter cassettes (MPSs) under JA, the bombarded leaves were kept in the Petri dishes with Hoagland solution supplemented with 50 µM MeJA (methyl jasmonate). After treatment, the plates were kept in the plant growth chamber at a temperature of 25°C, 16 h light/8 h dark period for 48 h. The transient expression studies using a biolistic system were performed as described by Mehrotra *et al.* (2005). In brief, treated leaves were incubated at 25°C and 16 h light/8 h dark photoperiod for 48 h. Subsequently, the leaves were immediately frozen, grounded in liquid nitrogen, and treated with GUS extraction buffer (50 mM Na_2_HPO_4_ pH 7.0, 1 mM EDTA, 0.1% v/v Triton X-100, 1.0 mM DTT and 0.1% SLS). The glucuronidase activity was assayed in cell-free extracts using 4-methyl umbelliferyl glucuronide (Jefferson *et al.* 1987). Relative fluorescence of 4-methylumbelliferone (MU) was determined using the Perkin Elmer Spectrofluorometer with excitation at 365 nm and emission at 455 nm. The expression data were analyzed statistically using a *t*-test.

### Cloning of full-length promoter and introduction of mutation in ACGT region

We cloned the full-length promoter of PP2C-like gene (Gene id: AT5G59220) (Supplementary Fig. 2) using the Forward primer (5′-CGTCTAGAAAGTATTCACGCACCAAGGT-3′) and reverse primer (5′-GCTCTAGAACAAACACACTCCATCAC-3′). The mutated construct (bold in Supplementary Table 2 and Supplementary Fig. 2) of it was cloned using site-directed primer: 5′-CCGGATCCATGAAAGTGATGACC*TAATTAGTTGTATTTATAG-3′. We carried out transient expression studies using the GUS as a reporter gene for the full-length promoter of the gene AT5G59220 and its mutated version where ACGT was mutated.

## Results

### AAAG _(N)_ ACGT orientation is preferred over ACGT _(N)_ AAAG orientation

**Fig. 1.**
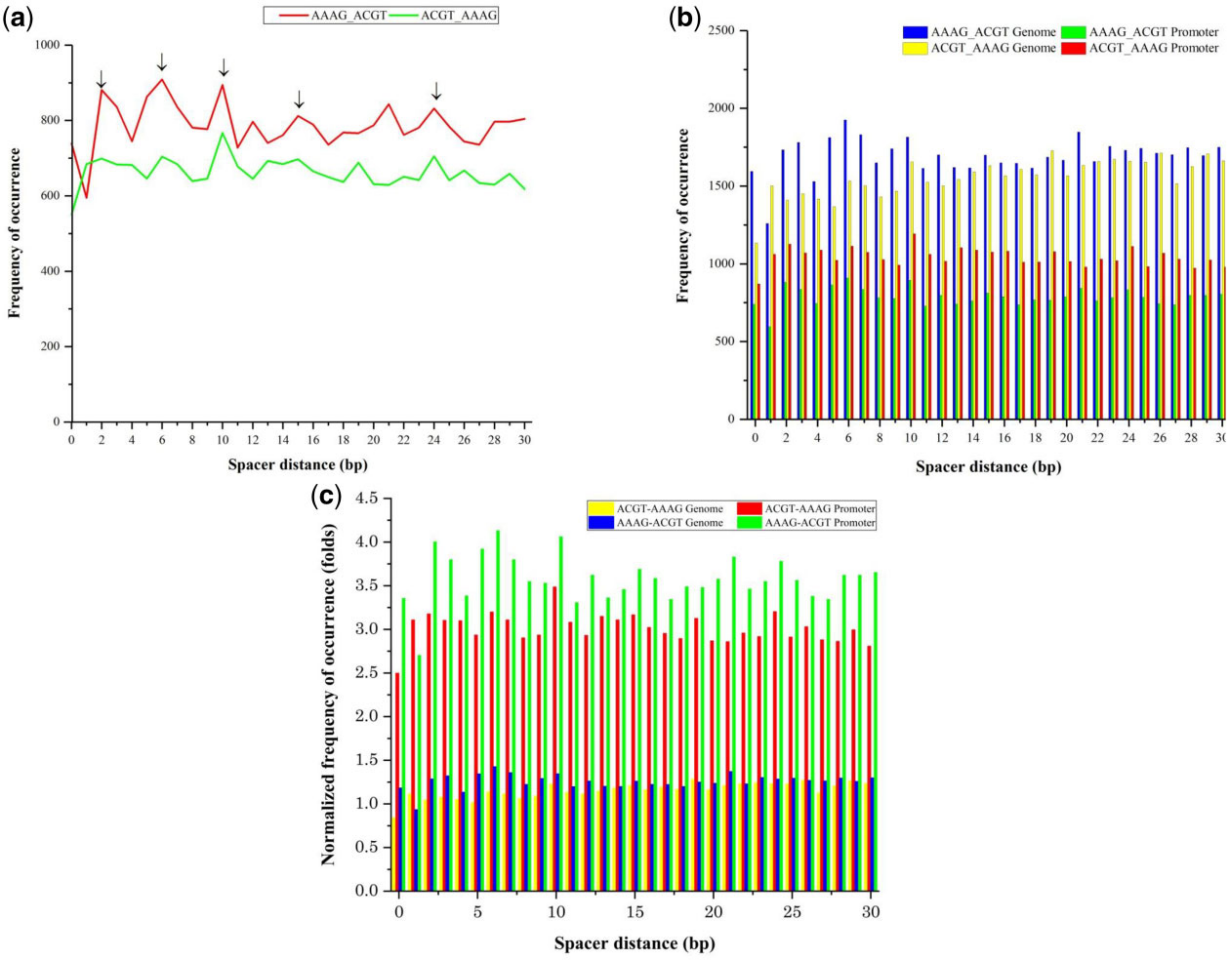
a) Frequency of occurrence of AAAG _(0–30 bp)_ ACGT and ACGT _(0–30 bp)_ AAAG motifs in tandem with varying spacer lengths in the promoters of *A. thaliana.* Arrows exhibit common peaks and dips. b) Comparison of frequency of occurrence of AAAG _(0–30 bp)_ ACGT and ACGT _(0–30 bp)_AAAG in the genome and promoter regions of *A. thaliana.* c) Normalized frequency of occurrence of AAAG _(0–30 bp)_ ACGT and ACGT _(0–30 bp)_ AAAG in the genome and promoter regions of *A. thaliana* showing the enrichment of motifs in promoters.

We performed an analysis of promoters from *A.* *thaliana* to determine the frequency of occurrence of AAAG and ACGT motifs in tandem with varying spacer lengths from 0 to 30 bp between them. Results indicated preferred orientation of AAAG _(__*N*__= 0__–__30 bp)_ ACGT over ACGT _(__*N*__= 0__–__30 bp)_ AAAG orientation (Fig. 1a). However, we also got similar results when the analysis was performed across the genome of *A. thaliana* (Fig. 1b).

### Enrichment of AAAG and ACGT motifs in promoters of *A. thaliana*

We found an average enrichment of 2 folds for most of the spacer length from 0 to 30 bp. We calculated the normalized frequency using the following formula :

$$=\frac{\text{Frequency of occurrence of the motif AAAG-ACGT or ACGT-AAAG (from 0 to 30 bp)}}{\text{Size of the genome}}$$

On normalizing the frequency of occurrence to the genome coverage we found an enrichment of around 2 folds in the promoter regions in AAAG-ACGT (range: 1.7–2.0) and ACGT-AAAG orientation (range: 1.5–2.0) (Fig. 1c).A Student *t*-test was conducted to test the significance of the results which showed that frequencies of occurrences are statistically significant (*P*-value ≤ 0.001, *t* = 4.832). At all spacer lengths, except *N* = 1, the frequency of occurrence of AAAG_(N)_ACGT orientation is more than ACGT_(N)_AAAG orientation. Moreover, it can be interpreted from Fig. 1a that at certain spacer lengths, there is a very high degree of correlation between the occurrence of common peaks and dips. Common peaks and dips were observed from spacer lengths 6–11, showing a good correlation between 2 orientations (Pearson correlation coefficient of 0.51). A paired student *t*-test was also performed between 2 spacer sequences from 6 to 11 which also showed the difference was significant (*P*-value = 0.0012). Apart from spacer lengths 6**–**11, common peaks were observed at *N* = 15 and 24, and a common dip was observed at *N* = 25. For the orientation AAAG _(N)_ ACGT, the highest frequency of occurrence was at spacer lengths of *N* = 6, 10, and lowest for *N* = 1. Similarly, for ACGT _(N)_ AAAG orientation, the highest frequency of occurrence was observed at a spacer length of *N* = 10, and lowest at *N* = 0 (Fig. 1a). Student 2-tailed *t*-test indicated the difference between the 2 orientations AAAG _(N)_ ACGT, ACGT _(N)_ AAAG was highly significant. The *t*-value at 30 df for the test samples was 3.73 while the t critical is 2.04. We also did analysis using flanking sites of AAAG and ACGT motifs with the following sequences TAAAG_(N)_GACGTC and GACGTC_(N)_TAAAG which revealed significant peaks (spacer = 2, 8, 10, 13, 15, 17, 21, 24, 29) (Supplementary Fig. 3). The higher frequencies at various spacer lengths indicate the importance of these spacers in the binding of TFs to these sites which we confirmed through TFBS analysis on 24 spacer lengths.

### Low preference for AAAG and ACGT motifs in tandem as compared to other random motifs in tandem

**Fig. 2.**
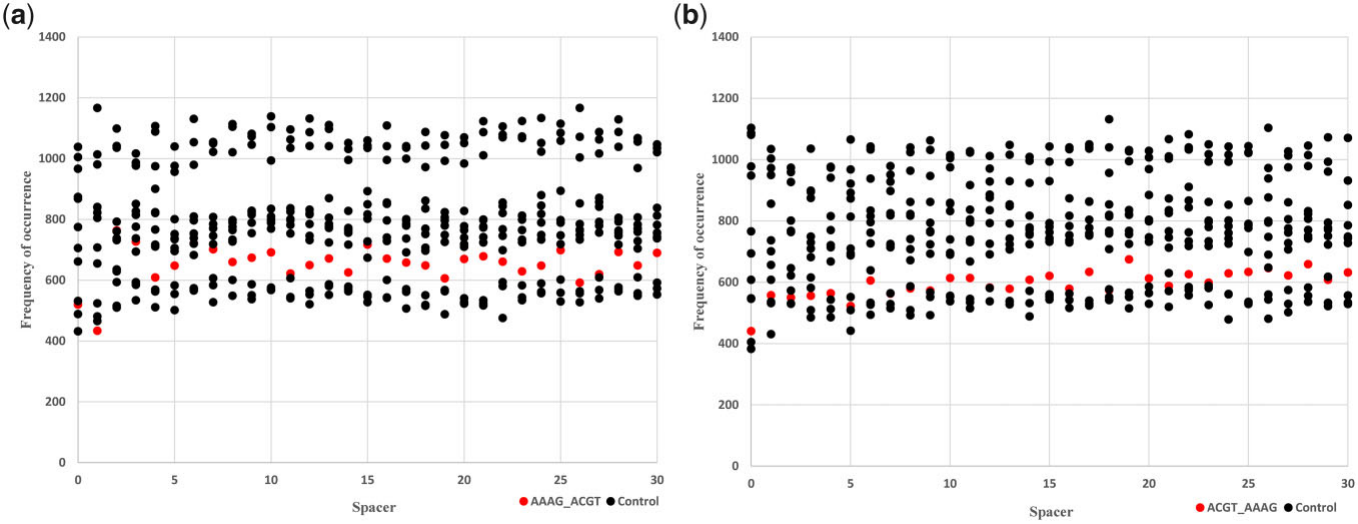
Comparison of frequency of occurrences of AAAG and ACGT motifs to random 4 bp control sequences. a) Comparison of frequency of occurrences of AAAG _(N)_ ACGT to random 4 bp control sequences. Frequency of occurrence of AAAG_(N)_ACGT and several control motifs shows limited preference for AAAG_(N)_ACGT motif as compared to random motifs. Red dots exhibit AAAG _(N)_ ACGT while black dots represent various control sequences. b) Comparison of frequency of occurrences of ACGT _(N)_ AAAG to random 4 bp control sequences. Frequency of occurrence of ACGT_(N)_AAAG and several control motifs shows limited preference for ACGT_(N)_AAAG motif as compared to random motifs. Red dots exhibit ACGT _(N)_AAAG, while black dots represent various control sequences.

Spacer sequence analysis and frequency of occurrence analysis were also conducted for some control motifs in tandem. Control motifs selected were AAGA, AGAA, and GAAA for AAAG generated by shuffling AAAG sequence in all possible ways likewise ACGT was shuffled to generate: CATG, GCAT, GTAC, and TCAG. It was ensured that control motifs are themselves not *cis-*regulatory elements and are completely random 4-nucleotide long sequences. The following charts (Fig. 2, a and b) show a comparison of frequencies of occurrence for AAAG and ACGT motifs in both orientations with control motifs from spacer lengths 0 to 30 bp. AAAG _(N)_ ACGT (Fig. 2a) and ACGT _(N)_ AAAG (Fig. 2b) both occur moderately less number of times as compared to control motifs, thereby, showing a limited preference for AAAG and ACGT motifs in tandem. Through paired student *t*-test we found statistically significant differences in these 2 orientations and among the control sequences only these AAAG _(N)_ GCAT, TCAG _(N)_ AGAA, are not statistically significant.

### “A” is the most conserved nucleotide in spacer sequences

The spacer sequences were obtained for all 0–30 spacer lengths for both orientations. Further, consensus sequences, based on threshold conditions for percentage A, T, G, C content (Mishra *et al.* 2009) were drawn for all spacer lengths for both possible orientations. It was observed that nucleotide “A” occurred the most number of times; highlighting the fact that “A” is the most conserved nucleotide in the consensus spacer sequences. In the orientation AAAG_(N)_ACGT, “A” nucleotide occurred at the first position in 14 spacer sequences (out of a possible 30), the third position in 4 spacer sequences, and at a few other positions in several other sequences. Whereas, the second most occurring nucleotide in consensus spacer sequences was “G” (Table 1). In the orientation ACGT_(N)_AAAG, nucleotide “G” occurred at the first position in a total of 5 spacer sequences: 1, 3, 6, 25, 27, while, nucleotide “A” occurred the most at 6 places, and nucleotide “T” at 1 instance (Table 2).

**Table 1.** Conserved sites in consensus spacer sequences for the orientation AAAG _(N)_ ACGT.

Spacer length (bp)	Position in consensus spacer sequence																													
	1	2	3	4	5	6	7	8	9	10	11	12	13	14	15	16	17	18	19	20	21	22	23	24	25	26	27	28	29	30
1	N																													
2	***G/C***	N																												
3	***G***	N	N																											
4	***G***	N	N	N																										
5	N	N	***G***	N	***C***																									
6	N	N	N	N	N	N																								
7	N	N	N	**A**	N	N	N																							
8	**A**	N	N	N	N	N	N	N																						
9	**A**	N	N	N	N	N	N	N	N																					
10	**A**	N	N	N	N	N	N	N	**A**	N																				
11	**A**	N	**A**	N	**A**	N	N	N	N	N	N																			
12	N	N	N	N	N	N	N	N	N	N	N	N																		
13	**A**	N	N	N	N	N	N	N	N	N	N	N	N																	
14	**A**	N	**A**	N	**A**	N	N	N	N	N	N	N	N	N																
15	**A**	N	N	N	N	N	N	N	N	N	N	N	N	N	N															
16	N	N	**A**	N	N	N	**A**	N	N	N	N	N	N	N	N	N														
17	N	N	N	N	N	**A**	N	N	N	N	N	N	N	N	N	N	N													
18	**A**	N	N	N	N	**A**	N	N	N	N	N	N	N	N	N	N	N	N												
19	**A**	N	N	N	N	N	N	N	N	N	N	N	N	N	N	N	N	N	N											
20	**A**	N	N	N	N	N	N	N	N	N	N	N	N	N	N	N	N	N	N	N										
21	N	N	N	**A**	N	N	N	N	N	N	N	N	N	N	N	N	N	N	N	N	N									
22	N	N	N	**A**	N	N	N	N	N	N	N	N	N	N	N	N	N	N	N	N	N	N								
23	N	N	N	N	N	**A**	N	N	N	N	N	N	N	N	N	N	N	N	N	N	N	N	N							
24	**A**	N	N	N	N	N	N	N	N	N	N	N	N	N	N	N	N	N	N	N	N	N	N	N						
25	N	N	N	N	N	N	N	N	N	N	N	N	N	N	N	N	N	N	N	N	N	N	N	N	N					
26	N	N	N	N	N	N	N	N	N	N	N	N	N	N	N	N	N	N	N	N	N	N	N	N	N	N				
27	**A**	N	N	N	N	N	N	N	N	N	N	N	N	N	N	N	N	N	N	N	N	N	N	N	N	N	N			
28	N	N	N	N	N	N	N	N	N	N	N	N	N	N	N	N	N	N	N	N	N	N	N	N	N	N	N	N		
29	N	N	**A**	N	N	N	N	N	N	N	N	N	N	N	N	N	N	N	N	N	N	N	N	N	N	**A**	N	**A**	N	
30	**A**	N	**A**	N	N	N	N	N	N	N	N	N	N	N	N	N	N	N	N	N	N	N	N	N	N	N	N	N	N	N

Nucleotides A and T are marked in bold, while nucleotides G and C are marked in bold italics.

**Table 2.** Conserved sites in consensus spacer sequences for the orientation ACGT_(N)_AAAG.

Spacer length (bp)	Position in consensus spacer sequence																													
	1	2	3	4	5	6	7	8	9	10	11	12	13	14	15	16	17	18	19	20	21	22	23	24	25	26	27	28	29	30
1	***G***																													
2	N	N																												
3	***G***	N	N																											
4	N	N	N	N																										
5	N	N	N	N	N																									
6	***G***	N	N	N	N	N																								
7	N	N	N	N	N	N	N																							
8	N	N	N	N	N	N	N	N																						
9	N	N	N	N	N	N	N	N	**A**																					
10	N	N	**A**	N	N	N	N	N	N	N																				
11	N	N	N	N	N	N	N	N	N	N	**A**																			
12	N	N	N	N	N	N	N	N	N	N	N	N																		
13	N	N	N	N	N	N	N	N	N	N	N	N	N																	
14	N	N	N	N	N	N	N	N	N	N	N	N	N	**A**																
15	N	N	N	N	N	N	N	N	N	N	N	N	N	N	N															
16	N	N	N	N	N	N	N	N	N	N	N	N	N	N	N	N														
17	N	N	N	N	N	N	N	N	N	N	N	N	N	N	N	N	N													
18	N	N	N	N	N	N	N	N	N	N	N	N	N	N	N	N	N	N												
19	N	N	N	N	N	N	N	N	N	N	N	N	N	N	N	N	N	N	N											
20	N	N	N	N	N	N	N	N	N	N	N	N	N	N	N	N	N	N	N	N										
21	N	N	N	N	N	N	N	N	N	N	N	N	N	N	N	N	N	N	N	N	N									
22	N	N	N	N	N	N	N	N	N	N	N	N	N	N	N	N	N	N	N	N	N	**A**								
23	N	N	N	N	N	N	N	N	N	N	N	N	N	N	N	N	N	N	N	N	N	N	N							
24	N	N	N	N	N	N	N	N	N	N	N	N	N	N	N	N	N	N	N	N	N	N	N	N						
25	***G***	N	N	N	N	N	N	N	N	N	N	N	N	N	N	N	N	N	N	N	N	N	N	N	N					
26	N	N	N	N	N	N	N	N	N	N	N	N	N	N	N	N	N	N	N	N	N	N	N	N	**A**	N				
27	***G***	N	N	N	N	N	N	N	N	N	N	N	N	N	N	N	N	N	N	N	N	N	N	N	N	N	N			
28	N	N	N	N	N	N	N	N	N	N	N	N	N	N	N	N	N	N	N	N	N	N	N	N	N	N	N	N		
29	N	N	N	N	N	N	N	N	N	N	N	**T**	N	N	N	N	N	N	N	N	N	N	N	N	N	N	N	N	N	
30	N	N	N	N	N	N	N	N	N	N	N	N	N	N	N	N	N	N	N	N	N	N	N	N	N	N	N	N	N	N

Most conserved nucleotides A and T are marked in bold, while most conserved nucleotides G and C are marked in bold italics.

### A 24-bp spacer sequence *TTGGGCTTTCAAAATTGTTAACTC* between AAAG and ACGT has the maximum number of TF binding sites

TF binding sites on highly occurring spacer sequences, along with MPS were determined using ConSite software as described in the *Materials* *and* *Methods* section. 139-nucleotide long MPS was observed to have only 27 plant-specific TF binding sites for 9 TFs: Agamous-like MADS-box protein (AGL3)** **–2, *A.* *thaliana* homeobox-leucine zipper protein (Athb-1)–2, *A.* *thaliana* homeobox-leucine zipper protein (Athb)-5–2, and DOF3–1, Gibberellin- and abscisic acid-regulated MYB (GAMYB)–5, High mobility group box 1 protein (HMG-1)–6, HMG-I/Y–6, Avian myeloblastosis virus MYB TF from Petunia hybrida (MYB.ph3)–1, and SQUAMOSA (SQUA)–2.

A 24-nucleotide long spacer sequence between AAAG and ACGT–AAAG***TTGGGCTTTCAAAATTGTTAACTC***ACGT, was reported to have 43 TF binding sites, a total of 16 additional sites than MPS: Athb-1–1, DOF2–2, DOF3–4, HMG-1–1, and 2 each of Maize nuclear factor binding protein 1A (MNB1A), MYB.ph3, Prolamine-box binding factor (PBF), and SQUA. In the other orientation, a 14-nucleotide long spacer sequence, ACGTGGATGCTATTAT TAAAAG, was reported to have the maximum number of TF binding sites at 39 sites, a total of 12 more than MPS-1 sites of SQUA, and 1 site each for AGL3, Athb-1, Athb-5, bZIP 910, DOF2, DOF3, MNB1A, MYB.ph3, and PBF. Many other sequences between spacer 2, 3, 4, 5, 6, 7, 10, 21, 23, 24, 29, 30 in AAAG_(N)_ACGT orientation while 0, 1, 2, 10, 14, 16, 17, 19, 26, 27, 29 in ACGT_(N)_AAAG orientation had more number of TF binding sites than MPS (Supplementary data sheet 1). Spacer 3, 4, 5, 7, 23, 24, 29 in AAAG _(N)_ ACGT orientation and spacer 0, 1, 10, 14, 16, 19, 26, 27, 29 had statistically significance than other sequences (Supplementary data sheet 1). This means that along with the distance between binding motifs there has been a selection for the sequence of the spacer in transcriptional regulation. We performed a similar TFBS analysis on *Glycine max* and found these TFs binding: AGL3, Athb-1, Athb-5, bZIP 910, bZIP 910, DOF2, DOF3, MNB1A, MYB.ph3, PBF, SQUA, Agamous, AGL3, GAMYB, HMG-IY, HMG-1, suggesting that the process of transcription regulation has essentially being conserved in eukaryotes (Supplementary data sheet 1).

### JA response is mediated by AAAG and ACGT repeat elements preferentially in an orientation independent manner

**Fig. 3.**
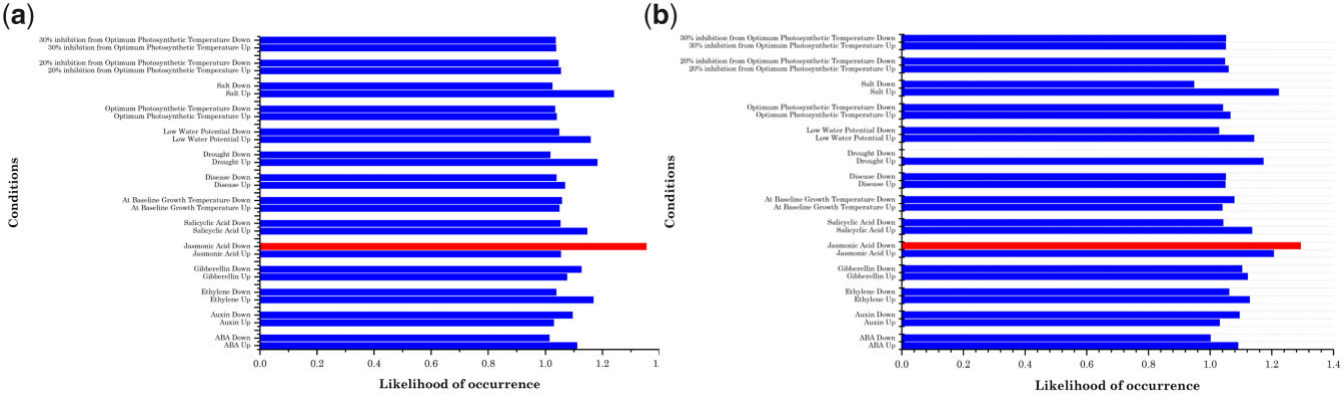
AAAG and ACGT containing promoters and their regulation under specific conditions. Using microarray-based expression data in *A. thaliana*, an analysis of promoters upregulated/downregulated/up-down regulated under 14 different conditions: environmental conditions (at baseline growth temperature, disease, drought, low water potential, at optimum photosynthetic temperature, salt, 20% inhibition from optimum photosynthetic temperature, 30% inhibition from optimum photosynthetic temperature) and hormones (ABA, auxin, ethylene, gibberellin, JA, SA) revealed downregulation of AAAG_(N)_ACGT containing promoters by JA. a) AAAG _(N)_ ACGT containing promoters and their regulation under specific conditions showing downregulation in response to JA. b) ACGT _(N)_ AAAG containing promoters and their regulation under specific conditions showing downregulation in response to JA.

We identified the genes upregulated, downregulated by specific environmental conditions, or by hormones containing an upstream AAAG _(N)_ ACGT and ACGT _(N)_ AAAG elements. By comparing genes regulated by a condition with genes containing multiple ACGT _(N)_ AAAG and AAAG _(N)_ ACGT elements, we calculated the likelihood of occurrence for each condition (Supplementary data sheets S2 and S3). We calculated the overall likelihood of occurrence for each condition, with a likelihood of 1 being that of random chance. Accordingly, conditions greater than 1.25 or above were selected. Results unveil that likelihood of occurrence value for AAAG_(N)_ACGT and ACGT_(N)_AAAG motif in promoters downregulated in response to JA is 1.29 and 1.35, respectively (Fig. 3, a and b). This points out that AAAG _(N)_ ACGT and ACGT _(N)_ AAAG being high in occurrence might play a crucial role in the promoters of genes downregulated during the JA response in both the orientation as their values are above the threshold by a significant margin. These observations suggest that AAAG and ACGT motifs are involved in the regulation of genes participating in abiotic and biotic stress conditions independent of their orientation. It was observed that the value of the likelihood of occurrence of none of the other conditions displayed any significant deviation.

### GO analysis reveals the presence of AAAG and ACGT in genes involved in ABA signaling, metal-ion binding, and transcription regulation

**Fig. 4.**
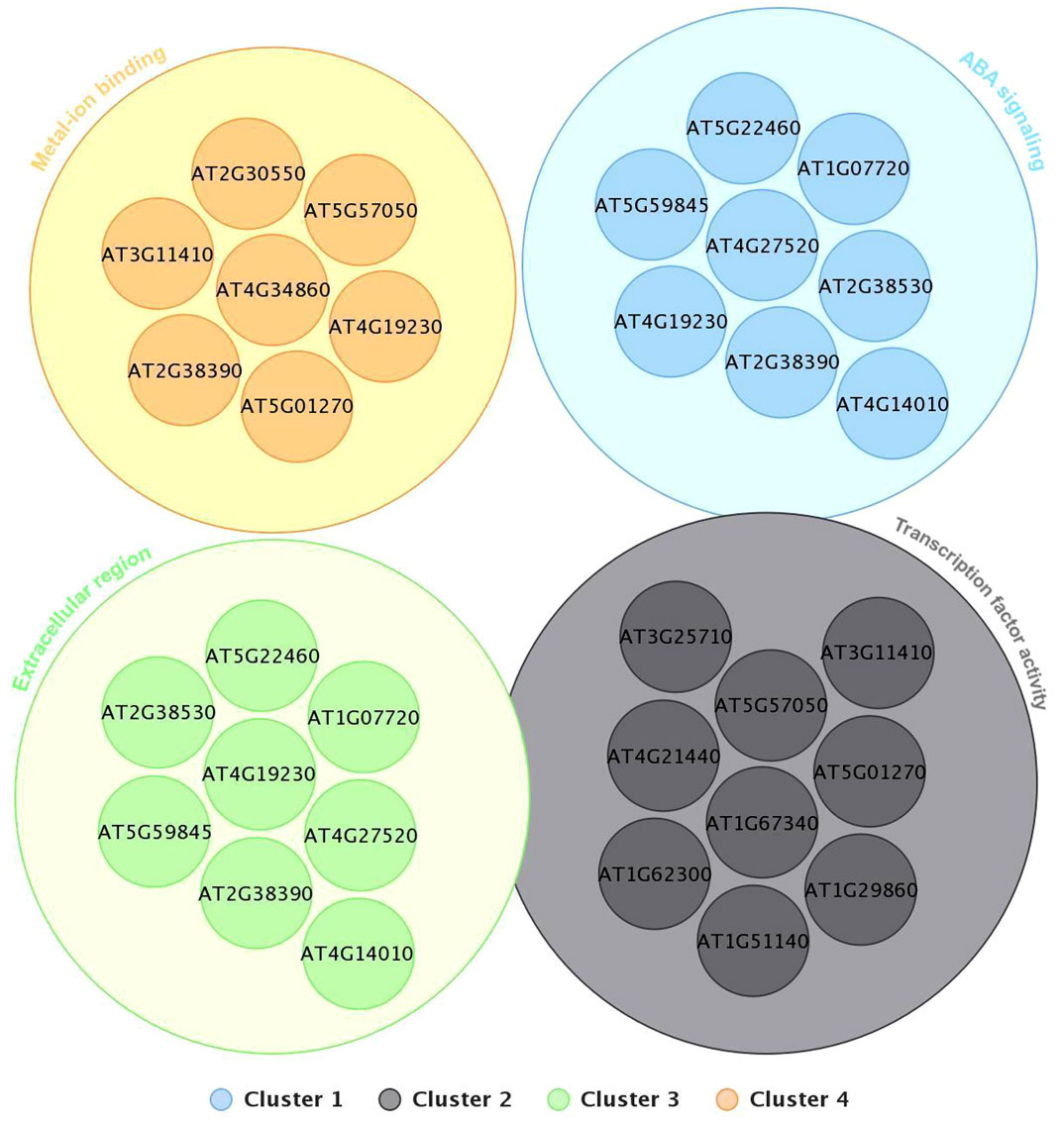
Cluster representation of JA downregulated genes with ACGT _(N)_ AAAG orientation based on functional characteristics. Cluster 1 represents genes involved with ABA signaling, cluster 2: TF activity, cluster 3: extracellular region, cluster 4: metal-ion binding.

GO term analysis revealed that among 34 genes with AAAG_(N)_ACGT orientation, 33 overlapping genes (97.1%) were of cellular components, 28 overlapping genes (82.4%) were found to be involved in biological processes and 24 overlapping genes (70.6%) were associated with molecular function in the cell. In the case of 33 genes with ACGT_(N)_AAAG orientation, all 33 genes (100%) were represented in cellular component, 24 overlapping genes (72.7%) were found to be associated with biological processes and 22 overlapping genes (66.7%) were found to be involved with molecular functions. We further refined the data by doing functional clustering on the data. Figure 4 represents 4 clusters for ACGT _(N)_ AAAG orientation. Terms attributed to ABA signaling, transcription regulation, extracellular region, and metal-ion binding were highly represented in the genes downregulated under JA with the ACGT-AAAG orientation with statistical significance. This reveals that all clusters are balanced since they have evenly distributed genes. The graph illustrates the respective gene in the clusters. Furthermore, each cluster was assessed using an Enrichment score (Huang *et al.* 2009a) (Supplementary Table 3). Overlapping gene ids among all clusters are shown in a 4-set Venn diagram (Supplementary Fig. 4). The cluster diagram shows 2 common genes (AT2G38390, AT4G19230) among clusters C1, C3, and C4, 3 common genes AT5G57050, AT5G01270, AT3G11410 between cluster 2 and 4 and C1 and C3 have 6 common genes AT2G38390, AT5G22460, AT4G19230, AT4G14010, AT4G27520, AT5G59845, AT2G38530, AT1G07720 while C2 and C4 have a total 6 and 2 unique genes respectively.

**Fig. 5.**
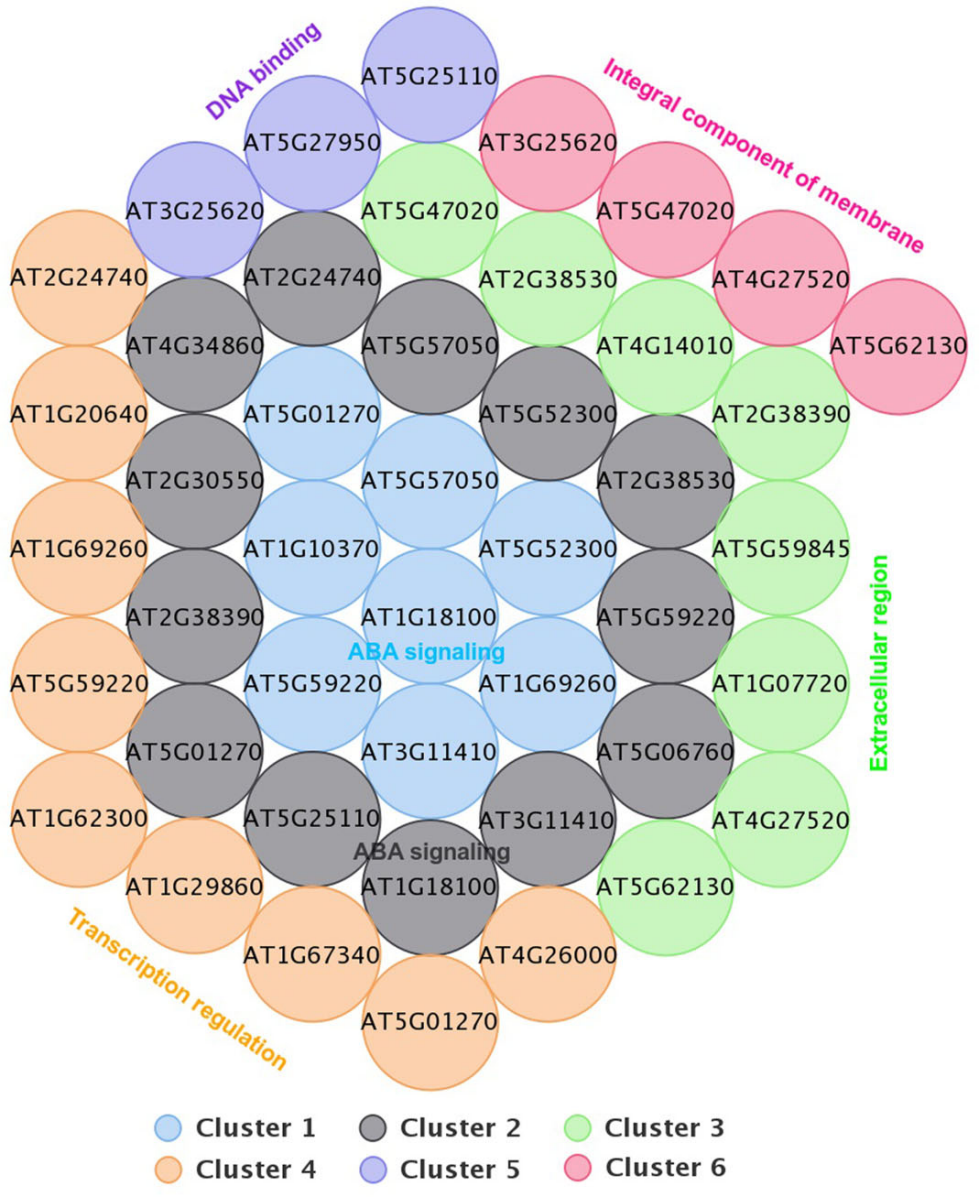
Cluster representation of JA downregulated genes with AAAG _(N)_ ACGT orientation based on function characteristics. Cluster 1 represents genes involved with ABA signaling; cluster 2: extracellular region; 3: transcription regulation; cluster 4: metal-ion binding; cluster 5: DNA binding; cluster 6: integral component of membrane.

Figure 5 represents the cluster diagram for the genes downregulated under JA with AAAG _(N)_ ACGT orientation. As the figure reveals, in AAAG _(N)_ ACGT orientation, out of 35 genes, no genes were identified as outliers and all of them were clustered into 6 groups. While clusters C1, C2, C3, and C4 are well-balanced (relatively evenly distributed), clusters C5 and C6 are significantly imbalanced consisting of 3 and 4 genes, respectively. In both the orientations of AAAG and ACGT [AAAG_(N)_ACGT and ACGT_(N)_AAAG], only 1 cluster was found to be significant with an enrichment score >2 (Supplementary Tables 3 and 4). This happened due to the overlapping properties among genes resulting in a soft clustering. A 6-set Venn diagram of the overlapping genes within clusters represents that every cluster has at least one overlapping gene with other clusters (Supplementary Fig. 5). However, no common gene has been found out among all the clusters. Cluster pairs of (C1, C2) have a total of 6 overlapping genes (AT5G01270, AT3G11410, AT5G59220, AT1G18100, AT5G57050, and AT5G52300) among which AT5G01270, AT5G59220 are also present in C4. In cluster pairs of C2, C3 there are 2 genes in common: AT2G38390, AT2G38530. Furthermore, other than the cluster triplets of (C1, C2, and C4) which have 2 genes in common (i.e. AT5G01270 and AT5G59220), no other triplets or larger intersection sets have a common gene. In both orientations, some genes were identified with 0 The individual clusters C1, C2, C3, C4, C5, and C6 have 1, 3, 3, 5, 1, and 0 unique genes.

**Fig. 6.**
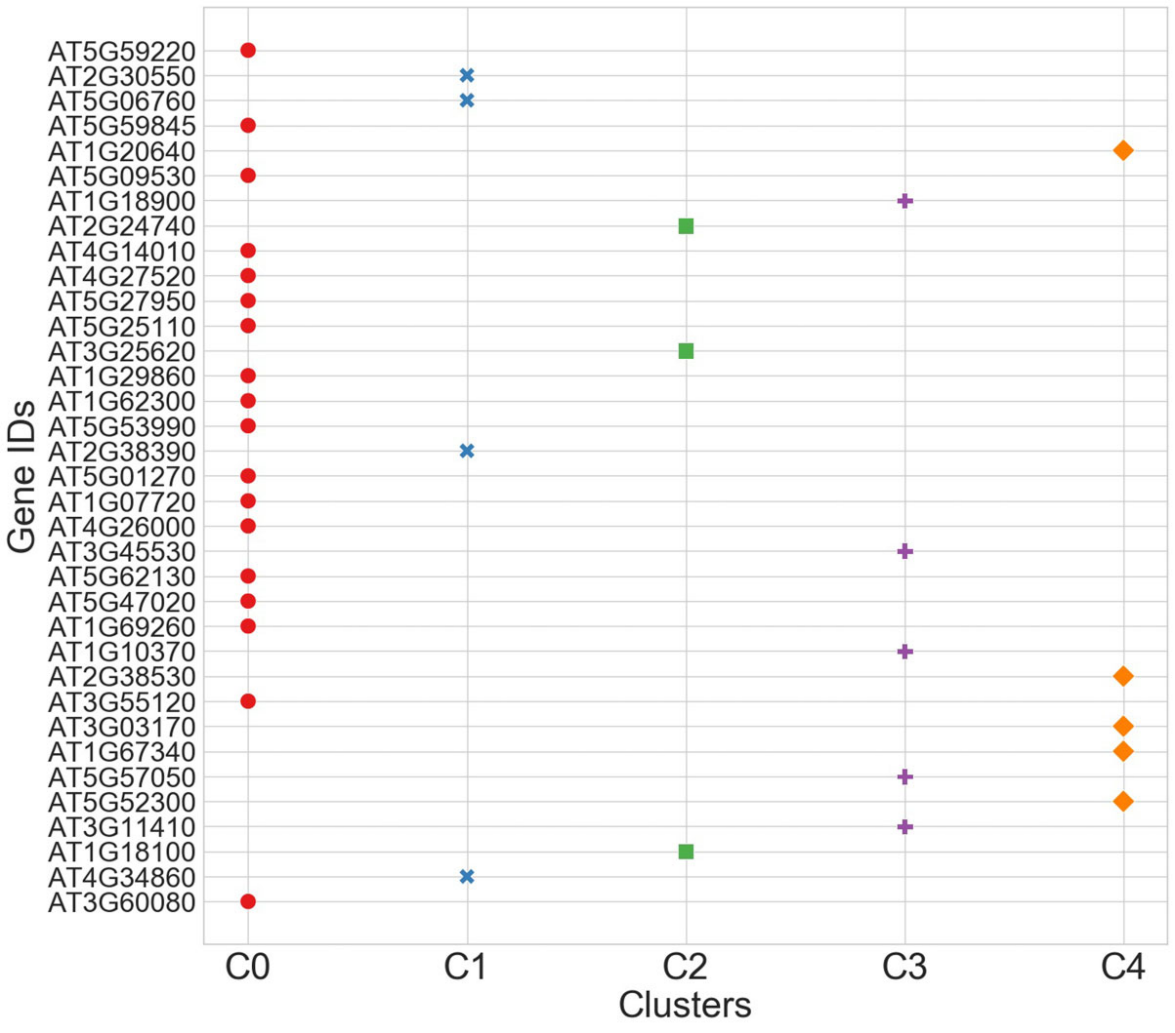
Scatter plot illustrating clustering of genes downregulated under JA response with AAAG _(N)_ ACGT orientation.

A larger Silhouette value indicates that clusters are significant. Silhouette score elbow curve for AAAG _(N)_ ACGT and ACGT _(N)_ AAAG orientations are shown in Supplementary Figs. 6 and 7 respectively. The optimal value of K for genes in AAAG _(N)_ ACGT orientation is 5 (Supplementary Fig. 6). Even though *K* = 7, 8, and 9, have higher Silhouette scores than *K* = 5 but those are bad picks for the optimal value of K due to wide fluctuation in the score. The fit time represents the time taken (in seconds) to fit the unsupervised model on the given data. Figure 6 shows the cluster representation of JA genes downregulated in AAAG _(N)_ ACGT orientation. Among 34 genes: 18, 4, 3, 5, and 5 genes were grouped in clusters 0, 1, 2, 3, and 4 respectively with a mean Silhouette score of 0.242. Figure 6 reveals that there are no outlier genes with any similarity from other genes. Each cluster has a minimum of 3 genes. Furthermore, except for cluster 0, all remaining clusters are well-shaped and equally distributed.

**Fig. 7.**
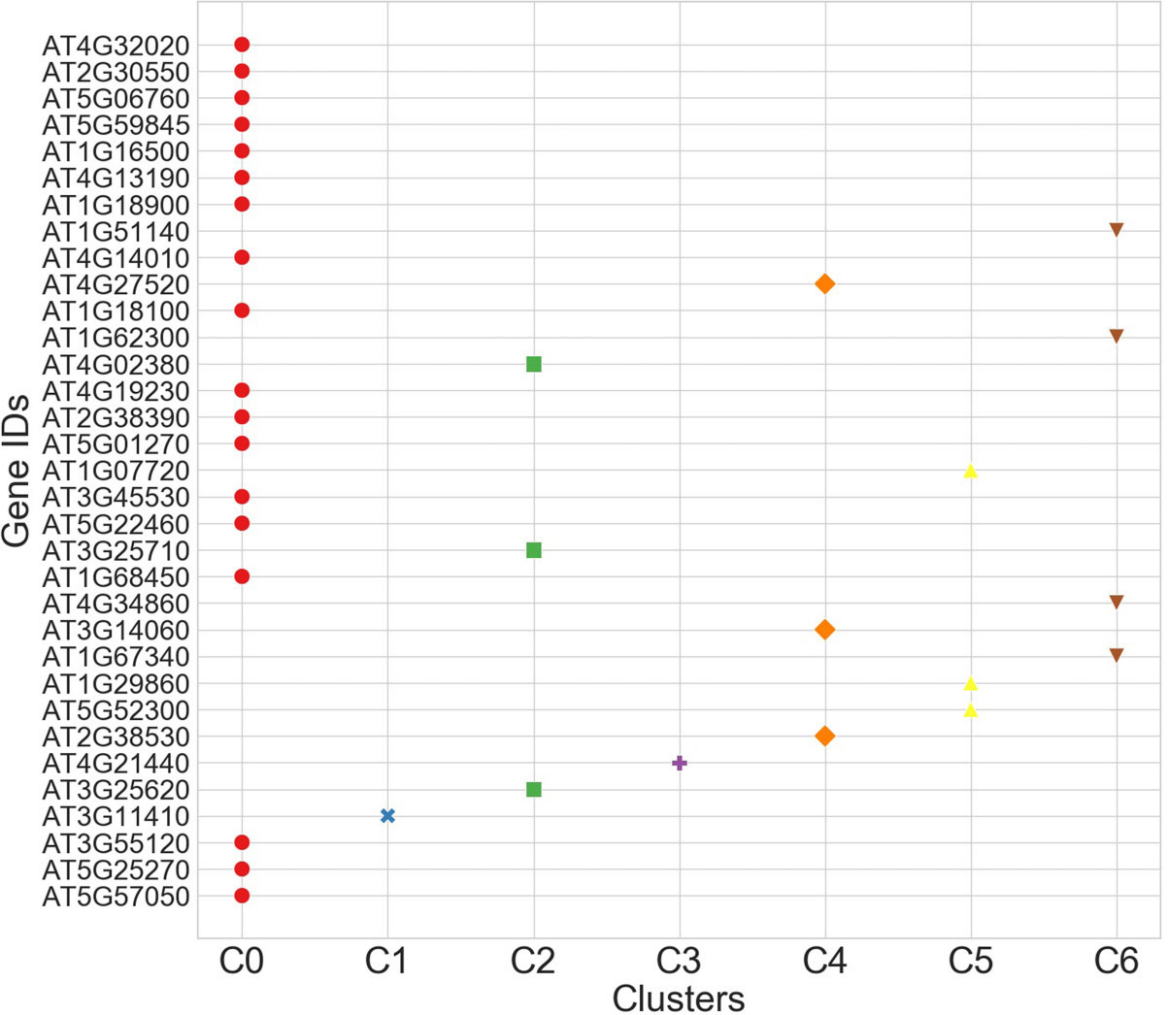
Scatter plot illustrating clustering of genes downregulated under JA response with ACGT _(N)_ AAAG orientation.

We found 7 optimal clusters in ACGT _(N)_ AAAG orientation (Supplementary Fig. 7). The Silhouette score is small due to the small number of resultant genes and sparse data with respect to 27 spacer values. The time graph shows that the algorithm took minimum time to fit the model for *K* = 7. Figure 7 shows the cluster representation of genes with respect to ACGT _(N)_ AAAG orientation. The graph reveals that the clusters C1 and C3 have only one gene (AT3G11410 and AT4G21440, respectively) making them an outlier. AT3G11410 has occurrences on a total of 5 spacers, i.e. 14, 16, 18, 20, and 22 whereas; AT4G21440 has occurrences on 13 and 22 spacer values. The graph reveals that cluster 0 has a maximum number of genes (18) having similar behavior evident for higher values of spacers. Similar to AAAG_(N)_ACGT orientation, 3 clusters are well-shaped and equally distributed identifying one gene as an outlier and the remaining genes as highly similar forming a dense cluster.

In order to gain insight into the regulatory functions of spacer sequences (0–30 bp) between the motifs, we identified the location of AAAG and ACGT motifs in both the orientation: AAAG _(__*N*__=__0__–__30)_ ACGT (Supplementary Table 5) and ACGT _(__*N*__=__0__–__30)_ AAAG (Supplementary Table 6) respectively on the promoters of the genes downregulated under JA response followed by their categorization according to the spacer lengths. For AAAG-ACGT orientation, 4 genes each were identified for the spacer 7 and 10. Group of the genes at spacer 7, are majorly cellular components. At spacer 10, 3 out of 4 genes are involved in the biological process with enriched term metabolic process.

For ACGT-AAAG orientation, we also got the highest frequency of occurrences i.e. 4 at the spacer length 20, 22, 23. GO term analysis reveals that these 4 genes at spacer length 20 are cellular components, enriched with the term metal-ion binding. Genes at spacer 22 are involved in biological processes, enriched with the term responses to osmotic stress. All 4 genes at spacer 23 are cellular components, enriched with the term nucleus.

### Transient expression analysis of AAAG and ACGT reporter cassette

The expression of the *gusA* gene in tobacco leaves bombarded with promoter-reporter cassette carrying a single AAAG and ACGT motif, in tandem and separated by a spacer of 5 and 25 nucleotides under JA was analyzed. The expression of the reporter gene driven by AAAG with ACGT motif separated by 5 and 25 nucleotides in both orientations was also studied. The leaves bombarded with a 50 + Pmec reporter cassette without any AAAG or ACGT motif were regarded as a control.

The data (as shown in Table 3) clearly shows a gradual increase in the reporter gene expression, expressed under the effect of a minimal promoter (50 + Pmec) carrying AAAG or ACGT activator motif as a single copy or 2, in tandem separated by a spacer length of 5, 25 in uninduced conditions. Under JA conditions, the constructs carrying (AAAG) (ACGT) in tandem or separated by a spacer length of 5, 25 decreased the reporter gene expression by 2.58, 2.82, 3.05 folds respectively. A similar trend of reduction was also observed for (ACGT) (AAAG) with the increasing spacer length of 0, 5, 25 bp by 2.34, 3.02, 3.48 folds, respectively, as compared to the control construct.

**Table 3.** Transient expression data showing promoter constructs carrying AAAG and ACGT motifs.

S.No.	Promoter cassette	**Uninduced (pmoles/min/mg protein** ± **SD)**	Fold activity as compared to 50 + Pmec	**JA (pmoles/min/mg protein** ± **SD)**	Fold induction	*P*-value
1	50 + Pmec	1,797 ± 26.8	1	1,805 ± 140	1.00	*P* = 0.477
2	(ACGT) + 50 + Pmec	2,683 ± 107	1.49	2,988 ± 122	1.11	*P* = 0.11
3	(AAAG) + 50 + Pmec	2,530 ± 95	1.40	3,013 ± 89	1.19	*P* = 0.06
4	(ACGT)_2_ + 50 + Pmec	3,208 ± 130	1.78	8,280 ± 137.2	2.58	***P* = 0.0008** ^b^
5	(AAAG)_2_ + 50 + Pmec	2,980 ± 99.8	1.65	7,623 ± 182	2.55	*P* = 0.01^a^
6	(ACGT)(AAAG) + 50 + Pmec	4,282 ± 102	2.38	1,829 ± 112	0.42	*P* = 0.013^a^
7	(AAAG)(ACGT) + 50 + Pmec	4,462 ± 119	2.48	1,726 ± 112	0.38	*P* = 0.0134^a^
8	(ACGT)_N5_ (AAAG) + 50 + Pmec	5,999 ± 180.22	3.33	1,983 ± 169	0.33	*P* = 0.013^a^
9	(AAAG)_N5_ (ACGT) + 50 + Pmec	5,860 ± 129	3.26	2,072 ± 113	0.35	*P* = 0.010^a^
10	(ACGT)_N25_ (AAAG) + 50 + Pmec	6,300 ± 228	3.50	1,807 ± 89	0.28	*P* = 0.012^a^
11	(AAAG)_N25_ (ACGT) + 50 + Pmec	5,900 ± 193	3.28	1,930 ± 118	0.32	*P* = 0.012^a^

a Indicates *P*-value < 0.05 and ^b^indicates *P*-value < 0.001.

### GUS activity of the protein phosphatase 2C (PP2C)-like promoter (AT5G59220) under JA response in tobacco leaves

Fluorometric assay was performed for the measurement of GUS activity. The expression of GUS in the full-length PP2C-like promoter (AT5G59220) was found to be high in uninduced conditions while in response to MeJA, approximately ∼6.5-fold decrease in GUS expression was observed (4048 ± 286) (Table 4). However, in the mutated construct, the readings are comparable in both the uninduced (18,000 ± 768) and induced (16,072 ± 207) condition revealing a similar yet not significant expression.

**Table 4.** Transient expression data of the promoter-reporter constructs.

S. No.	Promoter cassette	**Uninduced (pmoles/min/mg protein** ± **SD)**	**JA (pmoles/min/mg protein** ± **SD)**	Fold reduction	*P*-value
1	Full-length promoter	26,000 ± 1038	4,048 ± 286	6.4	0.001^a^
2	Mutated construct	18,000 ± 768	16,072 ± 207	5.85	0.07

a Indicates *P*-value < 0.001.

## Discussion

*Cis-*regulatory elements, AAAG and ACGT are known to play a vital role in regulating several processes including floral development (Rojas-Gracia *et al.* 2019), gibberellin response (Washio 2001), light response (Yanagisawa and Sheen 1998), tissue-specific expression (Vicente-Carbajosa *et al.* 1997), biotic and abiotic stress responses (Le Hir and Bellini 2013; Mehrotra *et al.* 2013; Ma *et al.* 2015; Wang *et al.* 2017). Previously we have done analysis on ACGT (Mehrotra *et al.* 2013) and AAAG (Mehrotra *et al.* 2014) *cis*-elements separately. In this present analysis, we have taken both the elements together with an attempt to decipher their interaction if any in stress response as these 2 *cis*-regulatory elements are known to interact with each other synergistically to regulate the expression of certain genes in specific stress conditions and also in the expression of seed storage protein genes (Singh 1998). Analyses performed on the extracted data revealed AAAG _(N)_ ACGT as the preferred orientation of these motifs for TF binding. An interesting observation was that the spacer lengths 6 and 10 occur at a maximum frequency in AAAG_(N)_ACGT orientation, while spacer length 10 also occurs maximally in ACGT_(N)_AAAG orientation. These sequences occur more than the other spacer sequences suggests that these lengths might serve as optimal lengths for TF binding (Kim and Maniatis 1997) also observed from TF binding site analysis (Supplementary data sheet 1). This indicates that along with the distance between motifs there has been a selection for the sequence of the spacer in transcriptional regulation. Further, 2 orientations show a correlation in peaks and dips for certain consecutive spacer lengths, as mentioned above, also point to the reversibility of TF binding or coevolution because of the high synergistic functionality of the 2 motifs (Yamamoto *et al.* 2006). Also, a probable reason for the dips could be that certain lengths might cause steric hindrance in the binding of TFs explaining the reason for the lower preference for certain specific sequences despite an optimal length (Yamamoto *et al.* 2006; Spitz and Furlong 2012). We found 1.5–2.0 folds enrichment of AAAG and ACGT motifs in promoters of *A. thaliana* as compared to its genome. On comparing the occurrence of 2 motifs—AAAG and ACGT in tandem with the other control sequences in the promoter region, it was revealed that these 2 motifs in tandem are less preferred than most of the control cases (Supplementary data sheet 1). From spacer sequence analysis, consensus sequences were drawn for each spacer length in both possible orientations. Consensus sequences showed a high degree of conservation of nucleotide “A.” In addition to this, “A” also occurred at the first position in 14 spacer sequences in the orientation AAAG _(N)_ ACGT. Also, “A” was found at the last position in the spacer sequence in 4 spacer lengths. These observations are in synchronization with the other inferences as AAAGA and AAAAG act as binding sites for several Dof family of TFs (Lijavetzky *et al.* 2003). Further, it was revealed that nucleotide “G” is preferred at the first position in 4 spacer sequences in the orientation ACGT _(N)_ AAAG.

Microarray analysis was carried out to determine the function of these motifs in upregulated or downregulated genes under environmental conditions and in response to phytohormones. Since AAAG and ACGT motifs are enriched in the genes getting downregulated in response to JA it could be deduced that these motifs are involved in the downregulation of genes taking part in JA responses in an orientation independent manner. The data suggest that the binding of the repressor seems to be distance-dependent rather than sequence-dependent so as to bring downregulation under JA response in an orientation-independent manner (Teif and Rippe 2011). GO analysis followed by cluster analysis revealed that the genes downregulated under JA conditions with AAAG and ACGT motifs in both the orientations are associated mainly with biological functions (ABA signaling, transcriptional regulation) cellular component (extracellular region) and molecular functions (metal-ion binding).

Cluster analysis on genes downregulated under JA with AAAG _(N)_ ACGT and ACGT_(N)_ AAAG motifs were conducted for all spacer lengths. In ACGT_(__N)_ AAAG orientation, cluster 0 has the maximum number of genes. These genes are mostly present on one spacer only. For example, AT5G22460, AT3G45530, AT5G01270, AT2G38390, AT4G19230, AT1G18100, AT4G14010, AT1G18900, AT4G13190, AT1G16500, AT5G59845, AT5G06760, AT2G30550, and AT4G32020 are present only on 7, 30, 2, 25, 27, 24, 12, 18, 4, 10, 26, 22, 21, and 11 respectively. Cluster 0 also has 4 genes (AT5G57050, AT5G25270, AT3G55120, and AT1G68450) which are present in more than 1 spacer but have similar spacers as other genes in their cluster. Surprisingly, there is no cluster that has genes all present on only 1 spacer length. We found an interesting insight that all the IDs present in cluster 2 (AT3G25620, AT3G25710, and AT4G02380) are present on spacer lengths 9 and 29 with 1 overlapping gene. AT3G11410 and AT4G21440 are identified as outliers in clusters 1 and 3 since these are the only genes with unique behavior. AT3G11410 appears on 5 spacer lengths while AT4G21440 appears twice on 1 spacer itself. In AAAG _(__N)_ ACGT orientation, cluster 1 has the maximum number of genes grouped together i.e. 18. All the genes are present on at least 1 spacer while only 2 IDs are present on 2 spacers i.e. AT3G60080 (spacer 3 and 19), AT3G55120 (spacer 3 and 18). In cluster 2, all genes (AT1G18100, AT3G25620, and AT2G24740) are present on exactly 1 spacer length, i.e. 15 and do not appear anywhere else. In cluster 4, all the genes except AT1G20640 are present on 2 spacers and share a common spacer too. For example, while AT1G67340, AT3G03170, AT1G20640 are present on spacer length 2, AT3G03170 if further present with AT5G52300 and AT2G38530 at spacer 28. Except for AT5G57050 AT4G34860, all IDs in cluster 3: AT3G11410, AT1G10370, AT3G45530, AT1G18900 are present on spacer 7 while AT5G57050 is present on spacer 29 and 30. Similarly, except AT4G34860, all gene ids in cluster 1: AT2G38390, AT5G06760, AT2G30550 are present at 10 spacers while AT4G34860 is also present at spacer 20. This analysis reveals that most of the genes in the clusters belong to a particular spacer length suggesting the importance of spacer length between AAAG and ACGT in the promoters of the genes involved in the biological processes in plants or function as cellular components.

The transient expression studies strengthened our in silico findings that the AAAG and ACGT motifs downregulate the gene expression under JA responses irrespective of their orientation. The data further suggests that the AAAG and ACGT motifs might be acting as a negative regulator leading to reduced reporter gene expression in response to JA in (AAAG)_N5_(ACGT), (AAAG)_N25_(ACGT), (ACGT)_N5_(AAAG), (ACGT)_N5_(AAAG) constructs. Our transient expression studies on full-length PP2C- like promoter and its mutated version indicate that the region between AAAG (TGATG) ACGT of this promoter is responsible for downregulation under JA treatment. As the expression of any gene, largely relies upon the *cis*-regulatory elements arranged within the promoter regions, so in order to modulate the expression of the transgene, the role of AAAG and ACGT motifs as the negative regulator in JA responses could be taken into consideration in the designing of synthetic promoters. The results of this study can assist in the promoter designing, as spacer lengths and specific sequences between AAAG and ACGT motifs for binding are required for triggering a stress response (Mehrotra *et al.* 2017). Conservation of spacer lengths of sequences between motifs and their copy number is vital for promoter designing (Mehrotra *et al.* 2011, 2017; Dhatterwal *et al.* 2019).

## Conclusion

This study was aimed at analyzing patterns of ACGT and AAAG *cis-*regulatory elements in the *A.* *thaliana* genome. We established preferences for specific orientation, specific spacer lengths, and specific conserved nucleotides in the promoter region for the above-mentioned 2 motifs in tandem. Further, the interplay of these 2 motifs was observed to have an effect on downregulation of genes under JA responses in an orientation-independent manner. This information would be crucial for designing stress-inducible synthetic promoters for the development of highly productive and stress-resistant transgenic crops.

## Data availability

The data generated/analyzed during the study are available in the supplementary data sheets, supplementary figures, and supplementary tables.

Supplemental material is available at *G3* online.

ZHK performed the experiments and drafted the article. SD and MBM helped in in-silico and statistical analysis. SLB helped in editing the manuscript. SA performed clustering on gene data and generated inferences. DG helped in gene expression studies. RM conceived the original idea and SM investigated and supervised the findings of this work. All authors discussed the results and contributed to the final manuscript.

## Funding

RM and SM are thankful to the Department of Science and Technology (DST) Government of India under the Science and Engineering Research Board (SERB) scheme for financial support. This work was supported by the DST-SERB project bearing file number EMR/2016/002470 sanctioned by the government of India to SM and RM.

## Conflicts of interest

The authors declare that the research was conducted in the absence of any commercial or financial relationships that could be constructed as a potential conflict of interest.

## Supplementary Material

jkac057_Supplementary_Data_sheet_S1

jkac057_Supplementary_Data_sheet_S2

jkac057_Supplementary_Data_sheet_S3

jkac057_Supplementary_Figure_S1

jkac057_Supplementary_Figure_S2

jkac057_Supplementary_Figure_S3

jkac057_Supplementary_Figure_S4

jkac057_Supplementary_Figure_S5

jkac057_Supplementary_Figure_S6

jkac057_Supplementary_Figure_S7

jkac057_Supplementary_Table_S1

jkac057_Supplementary_Table_S2

jkac057_Supplementary_Table_S3

jkac057_Supplementary_Table_S4

jkac057_Supplementary_Table_S5

jkac057_Supplementary_Table_S6

jkac057_Supplementary_Figure_Legend
