# Supplementary figures and images for: Genome-wide analysis of AAAG and ACGT cis-elements in Arabidopsis thaliana reveals their involvement with genes downregulated under jasmonic acid response in an orientation independent manner
Source: G3 (Bethesda). 2022 Mar 18;12(5):jkac057. doi: 10.1093/g3journal/jkac057 (PMC9073683; doi:10.1093/g3journal/jkac057)

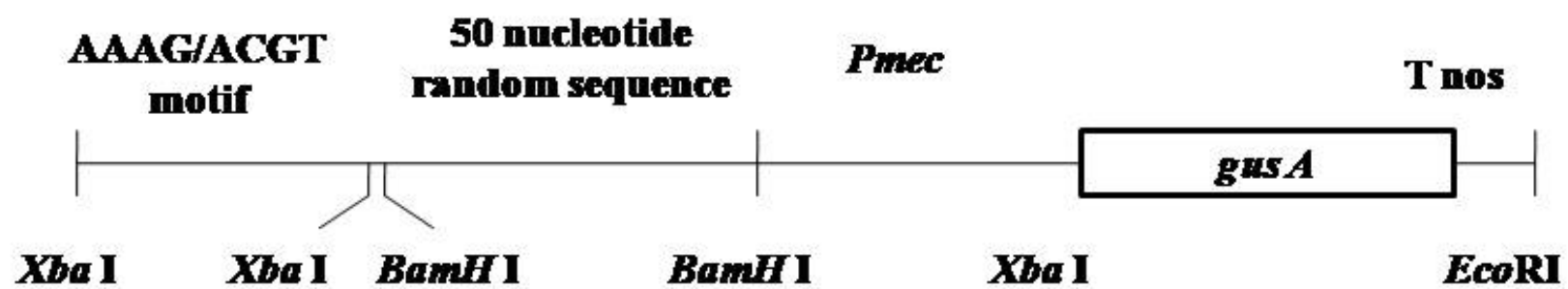

Supplement: jkac057_Supplementary_Figure_S1 [file jkac057_supplementary_figure_s1.pdf]

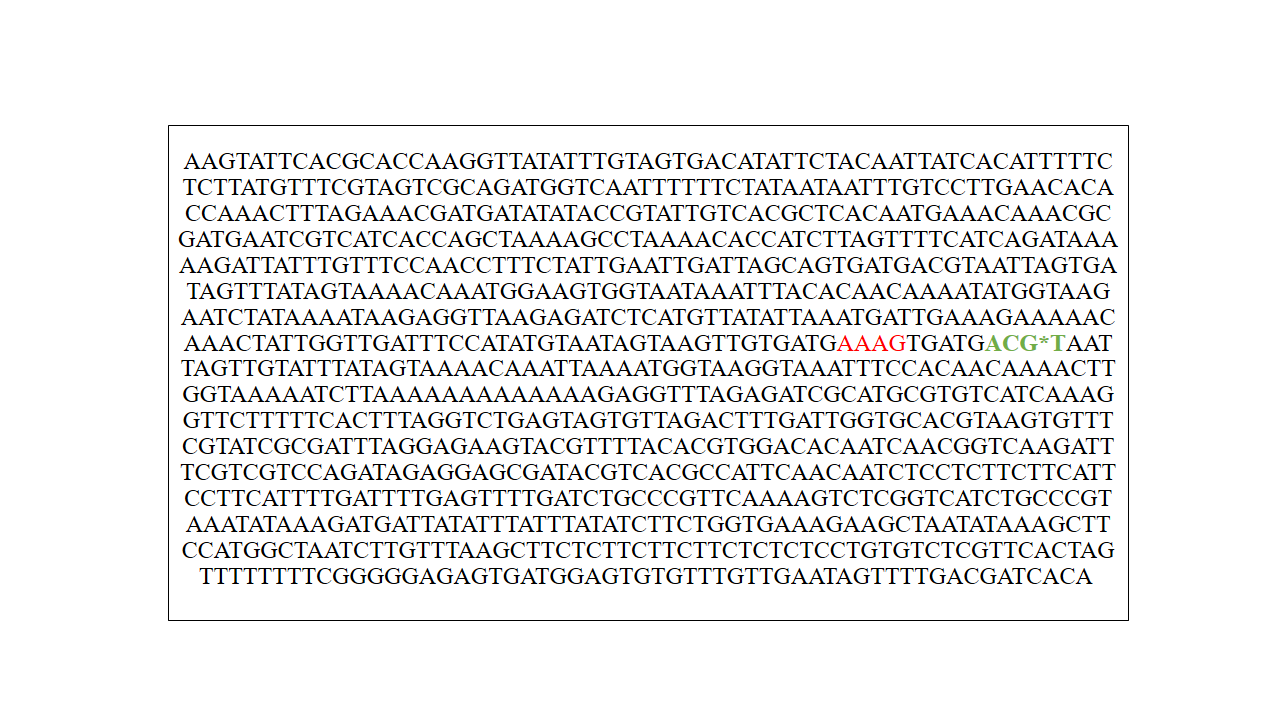

Supplement: jkac057_Supplementary_Figure_S2 [file jkac057_supplementary_figure_s2.zip › jkac057_Supplementary_Figure_S2.png]

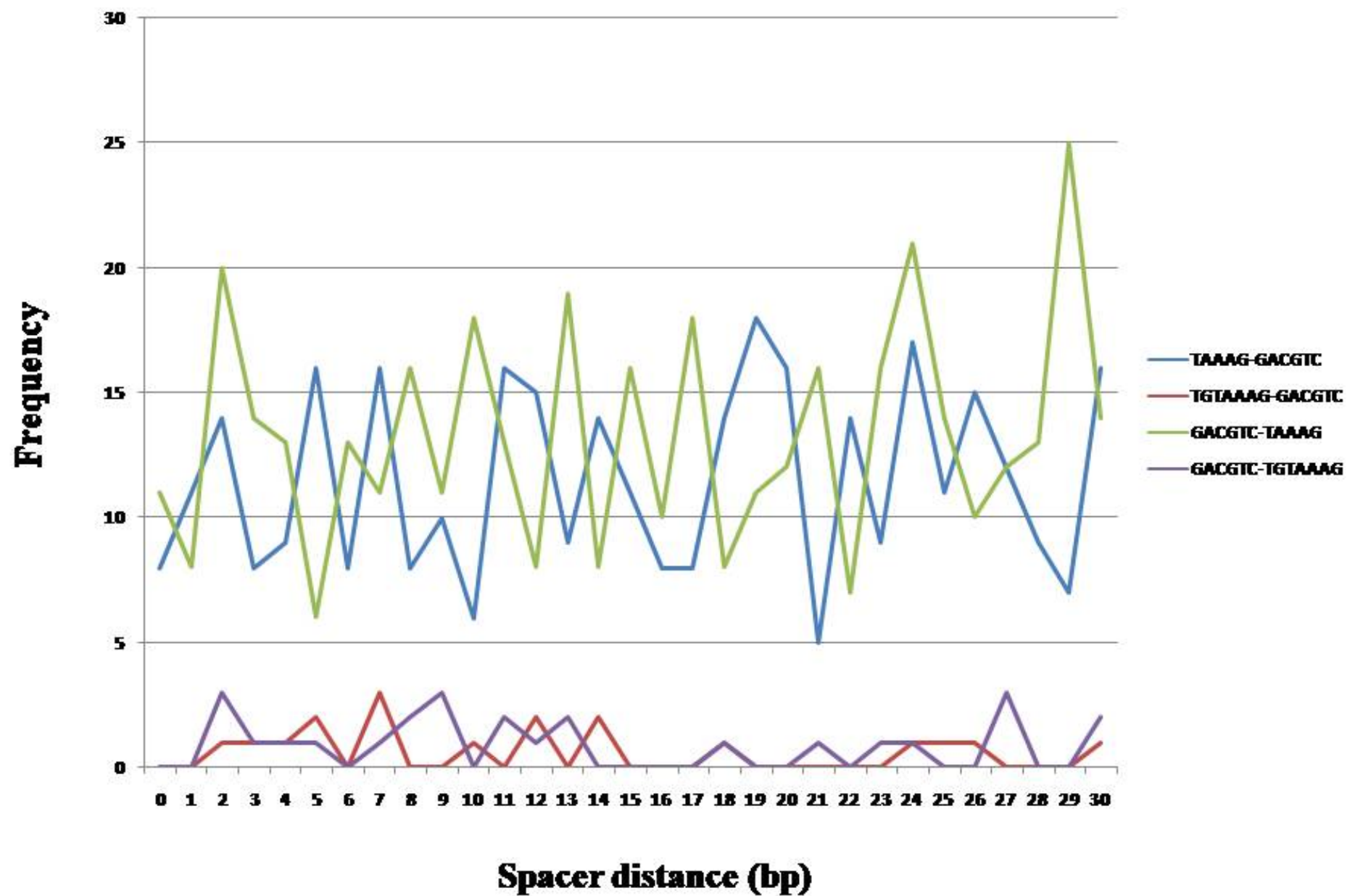

Supplement: jkac057_Supplementary_Figure_S3 [file jkac057_supplementary_figure_s3.pdf]

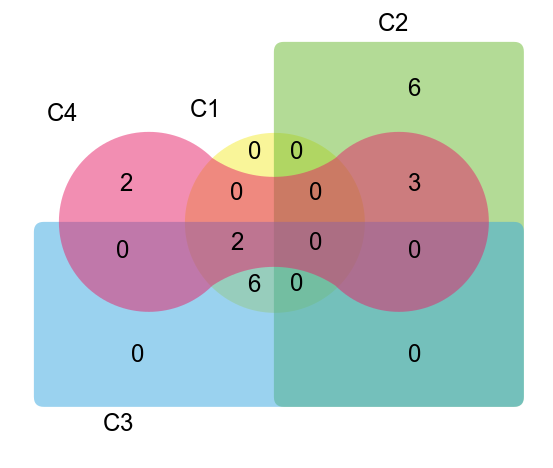

Supplement: jkac057_Supplementary_Figure_S4 [file jkac057_supplementary_figure_s4.zip › jkac057_Supplementary_Figure_S4.png]

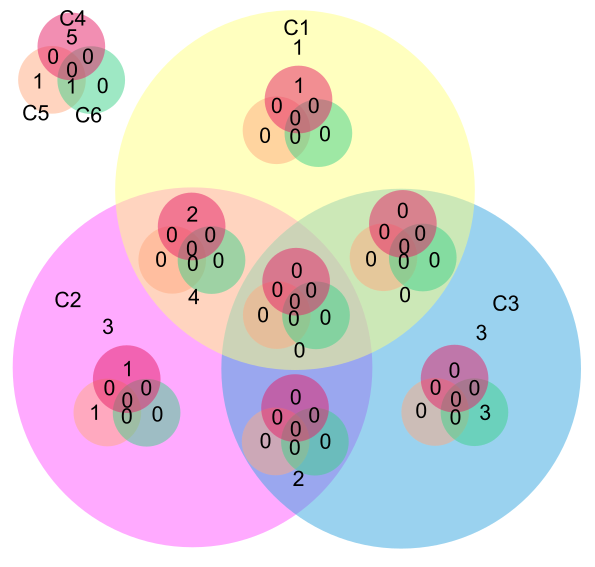

Supplement: jkac057_Supplementary_Figure_S5 [file jkac057_supplementary_figure_s5.zip › jkac057_Supplementary_Figure_S5.png]

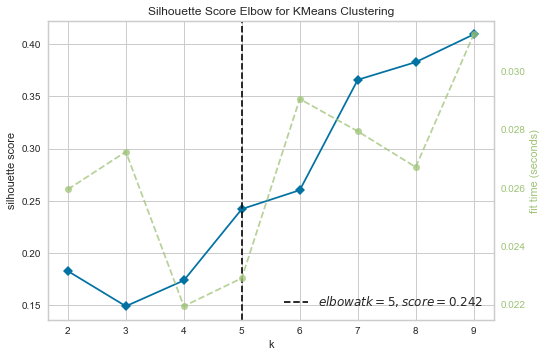

Supplement: jkac057_Supplementary_Figure_S6 [file jkac057_supplementary_figure_s6.zip › jkac057_Supplementary_Figure_S6.png]

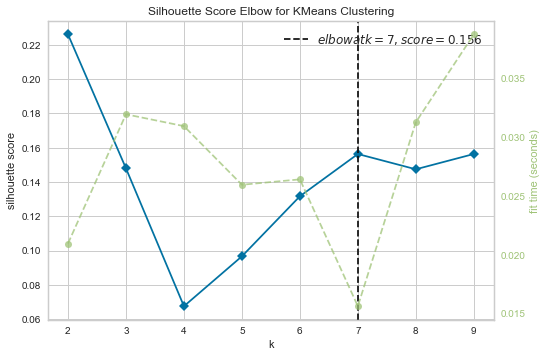

Supplement: jkac057_Supplementary_Figure_S7 [file jkac057_supplementary_figure_s7.zip › jkac057_Supplementary_Figure_S7.png]
