# Supplementary material for: Genome-wide analysis of AAAG and ACGT cis-elements in Arabidopsis thaliana reveals their involvement with genes downregulated under jasmonic acid response in an orientation independent manner
Source: G3 (Bethesda). 2022 Mar 18;12(5):jkac057. doi: 10.1093/g3journal/jkac057 (PMC9073683; doi:10.1093/g3journal/jkac057)
Supplement: jkac057_Supplementary_Table_S1 [file jkac057_supplementary_table_s1.docx]

**Supplementary Table S1: An illustration of promoter constructs of AAAG/ACGT motif separated by spacer sequences of varying lengths used for transient expression study.** The core motif AAAG/ACGT is shown in bold and the random sequence of 5 and 25 bp is underlined.

| **Constructs** | **Representation** |
| --- | --- |
| TCTAGA***ACGT***TCTAGA | (ACGT) |
| TCTAGA***AAAG***TCTAGA | (AAAG) |
| TCTAGA***ACGTACGT***TCTAGA | (ACGT)_2_ |
| TCTAGA***AAAGAAAG***TCTAGA | (AAAG)_2_ |
| TCTAGA***ACGTAAAG***TCTAGA | (ACGT)(AAAG) |
| TCTAGA***AAAGACGT***TCTAGA | (AAAG)(ACGT) |
| TCTAGA***ACGT*** GGCTA***AAAG***TCTAGA | (ACGT)_N5_(AAAG) |
| TCTAGA***AAAG*** GGCTA***ACGT***TCTAGA | (AAAG)_N5_(ACGT) |
| TCTAGA***ACGT***GGCTATGGCGGAGCAAGATTCACTC***AAAG***TCTAGA | (ACGT)_N25_(AAAG) |
| TCTAGA***AAAG***GGCTATGGCGGAGCAAGATTCACTC***ACGT***TCTAGA | (AAAG)_N25_(ACGT) |
