# Supplementary material for: Genome-wide analysis of AAAG and ACGT cis-elements in Arabidopsis thaliana reveals their involvement with genes downregulated under jasmonic acid response in an orientation independent manner
Source: G3 (Bethesda). 2022 Mar 18;12(5):jkac057. doi: 10.1093/g3journal/jkac057 (PMC9073683; doi:10.1093/g3journal/jkac057)
Supplement: jkac057_Supplementary_Table_S2 [file jkac057_supplementary_table_s2.docx]

**Supplementary Table S2: Promoter constructs for mutation studies. * indicates the base substitution (G to C)**

| **Constructs** | **Representation** |
| --- | --- |
| TCTAGA***AAAG***TTTAC***ACG*T***TCTAGA | (AAAG)_N5_(ACG*T) |
