# Supplementary material for: Genome-wide analysis of AAAG and ACGT cis-elements in Arabidopsis thaliana reveals their involvement with genes downregulated under jasmonic acid response in an orientation independent manner
Source: G3 (Bethesda). 2022 Mar 18;12(5):jkac057. doi: 10.1093/g3journal/jkac057 (PMC9073683; doi:10.1093/g3journal/jkac057)
Supplement: jkac057_Supplementary_Table_S3 [file jkac057_supplementary_table_s3.docx]

**Supplementary Table S3: Annotation clustering enrichment score for genomes in ACGT _(N)_ AAAG orientation**

| **Cluster** | **C1** | **C2** | **C3** | **C4** |
| --- | --- | --- | --- | --- |
| **Enrichment Score** | 3.368 | 0.586 | 0.526 | 0.467 |
