# Supplementary material for: Genome-wide analysis of AAAG and ACGT cis-elements in Arabidopsis thaliana reveals their involvement with genes downregulated under jasmonic acid response in an orientation independent manner
Source: G3 (Bethesda). 2022 Mar 18;12(5):jkac057. doi: 10.1093/g3journal/jkac057 (PMC9073683; doi:10.1093/g3journal/jkac057)
Supplement: jkac057_Supplementary_Table_S4 [file jkac057_supplementary_table_s4.docx]

**Supplementary Table S4: Annotation clustering enrichment score for genomes in AAAG _(N)_ ACGT orientation**

| **Cluster** | **C1** | **C2** | **C3** | **C4** | **C5** | **C6** | **Outlier** |
| --- | --- | --- | --- | --- | --- | --- | --- |
| **Enrichment Score** | 2.142 | 0.551 | 0.479 | 0.410 | 0.116 | 0.002 | NA |
