# Supplementary material for: Genome-wide analysis of AAAG and ACGT cis-elements in Arabidopsis thaliana reveals their involvement with genes downregulated under jasmonic acid response in an orientation independent manner
Source: G3 (Bethesda). 2022 Mar 18;12(5):jkac057. doi: 10.1093/g3journal/jkac057 (PMC9073683; doi:10.1093/g3journal/jkac057)
Supplement: jkac057_Supplementary_Table_S5 [file jkac057_supplementary_table_s5.docx]

**Supplementary Table 5 Position of AAAG and ACGT in promoters of the genes downregulated under jasmonic acid**

| S. No. | Gene | Description | Position of AAAG | Position of ACGT | Spacer length |
| --- | --- | --- | --- | --- | --- |
| 1 | AT3G11410 | *Arabidopsis thaliana* protein phosphatase 2CA, | 730  734  738 | 745  745  745 | 11  7  3 |
| 2 | AT3G60080 | RING/U-box superfamily protein | 601  837 | 624  844 | 19  3 |
| 3 | AT5G52300 | CAP160 protein (LTI65) | 873  885 | 905  905 | 28  16 |
| 4 | AT5G57050 | Protein phosphatase 2C family protein (ABI2) | 531  665 | 565  698 | 30  29 |
| 5 | AT1G67340 | HCP-like superfamily protein with MYND-type zinc finger | 512  529 | 535  535 | 19  2 |
| 6 | AT3G03170 | Hypothetical protein | 731  731 | 751  763 | 16  28 |
| 7 | AT3G55120 | Chalcone-flavanone isomerase family protein (TT5) | 891  906 | 913  913 | 18  3 |
| 8 | AT2G38530 | Lipid transfer protein | 719  749 | 725  781 | 2  28 |
| 9 | AT4G34860 | Plant neutral invertase family protein | 690  921 | 714  935 | 20  10 |
| 10 | AT1G10370 | Glutathione S-transferase family protein (ERD9) | 861  861 | 872  895 | 7  30 |
| 11 | AT1G69260 | ABI five binding protein (AFP1) | 668 | 683 | 11 |
| 12 | AT5G47020 | MraZ | 116 | 143 | 23 |
| 13 | AT5G62130 | Per1-like family protein; | 137 | 145 | 4 |
| 14 | AT3G45530 | Cysteine/Histidine-rich C1 domain family protein | 822 | 833 | 7 |
| 15 | AT4G26000 | RNA-binding KH domain-containing protein (PEP) | 914 | 940 | 22 |
| 16 | AT1G07720 | 3-ketoacyl-CoA synthase | 244 | 253 | 5 |
| 17 | AT5G01270 | Carboxyl-terminal domain (CTD) phosphatase-like | 681 | 698 | 13 |
| 18 | AT2G38390 | Peroxidase superfamily protein | 799 | 813 | 10 |
| 19 | AT5G53990 | UDP-Glycosyltransferase superfamily protein | 558 | 580 | 18 |
| 20 | AT1G62300 | WRKY family transcription factor(WRKY6) | 927 | 949 | 18 |
| 21 | AT1G29860 | WRKY DNA-binding protein 71(WRKY71) | 675 | 685 | 6 |
| 22 | AT1G18100 | PEP  (phosphatidylethanolamine-binding protein) | 835 | 854 | 15 |
| 23 | AT3G25620 | ABC-2 type transporter family protein | 147 | 166 | 15 |
| 24 | AT5G25110 | CBL-interacting protein kinase 25(CIPK25) | 47 | 76 | 25 |
| 25 | AT5G27950 | P-loop containing nucleoside triphosphate hydrolases superfamily protein | 339 | 349 | 6 |
| 26 | AT4G27520 | Early nodulin-like protein 2 | 854 | 871 | 13 |
| 27 | AT4G14010 | Ralf-like 32( RALFL32) | 618 | 651 | 29 |
| 28 | AT2G24740 | SET domain group 21(SDG21) | 231 | 250 | 15 |
| 29 | AT1G18900 | Pentatricopeptide repeat (PPR) superfamily | 552 | 563 | 7 |
| 30 | AT5G09530  (PELPK1, PRO-GLU-LEU\|ILE\|VAL-PRO-LYS 1, PROLINE-RICH PROTEIN 10, PRP10) | Hydroxyproline-rich glycoprotein family | 780 | 797 | 13 |
| 31 | AT1G20640 | Plant regulator RWP-RK family protein | 606 | 612 | 2 |
| 32 | AT5G59845 | Gibberellin-regulated family protein | 142 | 172 | 26 |
| 33 | AT5G06760 | Late Embryogenesis Abundant 4-5 (LEA4-5) | 860 | 874 | 10 |
| 34 | AT2G30550 | Alpha/beta-Hydrolases superfamily protein | 116 | 130 | 10 |
| 35 | AT5G59220 | Protein phosphatases type 2C). Functions as a negative regulator of osmotic stress and ABA signaling | 457 | 465 | 5 |
