# Supplementary material for: Genome-wide analysis of AAAG and ACGT cis-elements in Arabidopsis thaliana reveals their involvement with genes downregulated under jasmonic acid response in an orientation independent manner
Source: G3 (Bethesda). 2022 Mar 18;12(5):jkac057. doi: 10.1093/g3journal/jkac057 (PMC9073683; doi:10.1093/g3journal/jkac057)
Supplement: jkac057_Supplementary_Table_S6 [file jkac057_supplementary_table_s6.docx]

**Supplementary Table 6: Position of ACGT and AAAG in promoters of the genes downregulated under jasmonic acid**

| S.No. | Gene | Gene name | ACGT | AAAG | Spacer length |
| --- | --- | --- | --- | --- | --- |
| 1 | AT3G11410 | Protein phosphatase 2CA(PP2CA) | 712  712  712  745  745 | 730  734  738  765  769 | 14  18  22  16  20 |
| 2 | AT5G52300 | CAP160 protein (LTI65) | 278  278  955 | 285  299  978 | 3  17  19 |
| 3 | AT5G57050 | Protein phosphatase 2C family protein (ABI2) | 565  565  732 | 591  599  764 | 22  30  28 |
| 4 | AT4G21440 | MYB-like 102(MYB102) | 323  323  814 | 340  349  831 | 13  22  13 |
| 5 | AT5G25270 | Ubiquitin-like superfamily protein(AT5G25270) | 932  932 | 951  961 | 15  25 |
| 6 | AT1G29860 | WRKY DNA-binding protein 71(WRKY71) | 668  685 | 675  705 | 3  16 |
| 7 | AT3G25620 | ABC-2 type transporter family protein (ABCG21) | 166  243 | 199  256 | 29  9 |
| 8 | AT1G67340 | HCP-like superfamily protein with MYND-type zinc finger | 535  842 | 562  847 | 23  1 |
| 9 | AT2G38530 | Lipid transfer protein 2 (LTP2 | 614  725 | 636  749 | 18  20 |
| 10 | AT3G55120 | Chalcone-flavanone isomerase family protein (TT5) | 270  270 | 287  298 | 13  24 |
| 11 | AT3G14060 | Hypothetical protein | 495  896 | 518  920 | 19  20 |
| 12 | AT4G34860 | Plant neutral invertase family protein (A/N-InvB) | 1  891 | 28  921 | 23  26 |
| 13 | AT1G68450 | VQ motif-containing protein (PDE337) | 295  532 | 323  537 | 24  1 |
| 14 | AT3G25710 | Basic helix-loop-helix 32 (BHLH32) | 819 | 852 | 29 |
| 15 | AT5G22460 | Alpha/beta-Hydrolases superfamily protein | 665 | 676 | 7 |
| 16 | AT3G45530 | Cysteine/Histidine-rich C1 domain family protein | 528 | 562 | 30 |
| 17 | AT1G07720 | 3-ketoacyl-CoA synthase 3(KCS3) | 253 | 260 | 3 |
| 18 | AT5G01270 | Carboxyl-terminal domain (ctd) phosphatase-like 2(CPL2 | 890 | 896 | 2 |
| 19 | AT2G38390 | Peroxidase superfamily protein | 289 | 318 | 25 |
| 20 | AT4G19230 | Cytochrome P450, family 707, subfamily A, polypeptide 1(CYP707A1) | 483 | 514 | 27 |
| 21 | AT4G02380 | Senescence-associated gene 21(SAG21) | 315 | 328 | 9 |
| 22 | AT1G62300 | WRKY family transcription factor(WRKY6) | 861 | 888 | 23 |
| 23 | AT1G18100 | PEBP (phosphatidylethanolamine-binding protein) family protein (E12A11) | 807 | 835 | 24 |
| 24 | AT4G27520 | Early nodulin-like protein 2 (ENODL2) | 387 | 411 | 20 |
| 25 | AT4G14010 | Ralf-like 32(RALFL32) | 831 | 847 | 12 |
| 26 | AT1G51140 | Basic helix-loop-helix (bHLH) DNA-binding superfamily protein(FBH3) | 270 | 297 | 23 |
| 27 | AT1G18900 | Pentatricopeptide repeat (PPR) superfamily protein (AT1G18900) | 634 | 656 | 18 |
| 28 | AT4G13190 | Protein kinase superfamily protein (AT4G13190) | 649 | 657 | 4 |
| 30 | AT1G16500 | Filamentous hemagglutinin transporter(AT1G16500) | 205 | 219 | 10 |
| 31 | AT5G59845 | Gibberellin-regulated family protein(AT5G59845) | 172 | 202 | 26 |
| 32 | AT5G06760 | Late Embryogenesis Abundant 4-5(LEA4-5) | 874 | 900 | 22 |
| 33 | AT2G30550 | Alpha/beta-Hydrolases superfamily protein | 958 | 983 | 21 |
| 34 | AT4G32020 | Serine/arginine repetitive matrix-like protein(AT4G32020) | 130 | 145 | 11 |
