# Supplementary material for: Genome-wide analysis of AAAG and ACGT cis-elements in Arabidopsis thaliana reveals their involvement with genes downregulated under jasmonic acid response in an orientation independent manner
Source: G3 (Bethesda). 2022 Mar 18;12(5):jkac057. doi: 10.1093/g3journal/jkac057 (PMC9073683; doi:10.1093/g3journal/jkac057)
Supplement: jkac057_Supplementary_Figure_Legend [file jkac057_supplementary_figure_legend.docx]

**Supplementary Figures Legends**

**Supplementary Figure S1: Schematic representation of the promoter-reporter cassette used for transient expression studies.**

**Supplementary Figure S2: Full-length promoter sequence of the PP2C-like gene (AT5G59220) from *Arabidopsis thaliana***

**Supplementary Figure S3: Frequency of occurrence of flanking sites of AAAG and ACGT from 0 to 30 bp in the genome of *Arabidopsis thaliana.***The frequency of occurrence of GACGTC **_(N)_** TAAAGwas higher among all the other sequences with peaks at 2,8,10,13,15,17, 21,24,29.

**Supplementary Figure S4: Venn diagram illustrating the gene distribution in various clusters in ACGT _(N)_ AAAG orientation**

**Supplementary Figure S5: Venn diagram illustrating the gene distribution in various clusters in AAAG _(N)_ ACGT orientation**

**Supplementary Figure S6: Silhouette Score Elbow Curve of number of clusters for genes downregulated under jasmonic acid response in AAAG_(N)_ACGT orientation based on spacer characteristics**

**Supplementary Figure S7: Silhouette Score Elbow Curve of number of clusters for genes downregulated under jasmonic acid response in ACGT_(N)_AAAG orientation based on spacer characteristics**
